# Taxonomy and phylogeny of five novel *Trichoderma* (Hypocreaceae, Hypocreales) species isolated from decaying wood in China

**DOI:** 10.3897/mycokeys.133.187172

**Published:** 2026-05-27

**Authors:** Guang-Shuo Jin, Jing Ji, Gui-Long Zhao, Bo Liang, Xin-Ze Li, Yang Yu, Wen-Ying Zhuang, Yu Li, Rui-Qing Ji, Zhao-Xiang Zhu

**Affiliations:** 1 Engineering Research Center of Edible and Medicinal Fungi, Ministry of Education, Jilin Agricultural University, Changchun 130118, Jilin Province, China Institute of Microbiology, Chinese Academy of Sciences Beijing China; 2 College of Plant Protection, Jilin Agricultural University, Changchun 130118, Jilin Province, China College of Plant Protection, Jilin Agricultural University Changchun China; 3 State Key Laboratory of Mycology, Institute of Microbiology, Chinese Academy of Sciences, Beijing 100101, China Ministry of Education, Jilin Agricultural University Changchun China

**Keywords:** Ascomycota, Hypocreales, new species, phylogenetic analysis, taxonomy

## Abstract

A taxonomic study was conducted on *Trichoderma* species isolated from decaying wood in China. As a result, five novel species, *T. flavobritdaniae*, *T. foliatum*, *T. fulviclavum*, *T. liaoningense*, and *T. parvostromatum*, were identified, described in detail, and illustrated based on morphological characteristics and molecular data inferred from sequences of the second largest subunit of RNA polymerase II (*rpb*2) and the translation elongation factor 1-alpha (*tef*1-α). Among the five new species, *T. flavobritdaniae*, *T. fulviclavum*, and *T. parvostromatum* exhibit both asexual and sexual morphs, whereas *T. foliatum* and *T. liaoningense* are currently known only from their asexual morphs. Notably, *T. fulviclavum* produces clavate stromata but does not cluster with clavate-stromatic species traditionally assigned to the Viride clade. Phylogenetic analyses recovered all five taxa as well-supported, distinct lineages assigned to the Longibrachiatum, Harzianum, Psychrophilum, Semiorbis, and Brevicompactum clades. The discovery of these new species from China improves the understanding of *Trichoderma* diversity and biogeography and provides genetic resources with potential agricultural and industrial applications.

## Introduction

*Trichoderma* species are of considerable economic importance across agricultural, industrial, and environmental sectors. In agriculture, they are widely used as biofertilizers that improve soil structure and nutrient availability, thereby enhancing soil conditions for crop cultivation and supporting soil functions closely linked to carbon cycling (Mishra et al. 2024; Zhu et al. 2024; Ma et al. 2025; Shao et al. 2025). They are also commonly applied for the biocontrol of plant diseases owing to their capacity to suppress diverse phytopathogens through competition, antibiosis, and mycoparasitism (Guzmán-Guzmán et al. 2025; Li et al. 2025). In industry, *Trichoderma* represents an important source of enzymes—including cellulases, xylanases, and proteases—widely used in biorefining and food processing, and ongoing genetic optimization further expands its biotechnological utility (Pandey et al. 2024; Zhao et al. 2024; García-Latorre et al. 2025). In addition, many species promote plant growth by modulating phytohormone signaling and improving nutrient uptake, contributing to increased yield and crop quality (Alwadai et al. 2024). Beyond production systems, *Trichoderma* also shows strong potential for biodegradation and bioremediation by transforming recalcitrant pollutants and mitigating environmental contamination (De Lima et al. 2025). Accurate taxonomy is therefore fundamental for reliable identification and downstream applications of *Trichoderma*.

While the diverse economic value of *Trichoderma* species highlights their biotechnological significance, a comprehensive understanding of this genus requires robust taxonomic and phylogenetic investigations, especially concerning novel species isolated from unique ecological niches such as decaying wood. The genus *Trichoderma* Pers. (Hypocreaceae, Hypocreales, Sordariomycetes, Ascomycota) is a widely distributed genus of fungi that occurs predominantly in soil, decaying wood, and as endophytes within plant tissues (Zhu et al. 2017). Since its establishment by Persoon in 1794, approximately 560 *Trichoderma* species have been formally described worldwide to date (Liu et al. 2021; Thokala et al. 2021; Zheng et al. 2021; An et al. 2022; Cao et al. 2022; Pollard-Flamand et al. 2022; Zeng et al. 2022; Lagashetti et al. 2023; Liu et al. 2023; Nascimento Brito et al. 2023; Perera et al. 2023; Sousa et al. 2023; Ye et al. 2023; Zhao et al. 2023; Cao et al. 2024; Huang et al. 2024; Ma et al. 2024; Wei et al. 2024; Zeng et al. 2024; Rashad et al. 2025; Tan et al. 2025; Zhao et al. 2025). Due to its extensive geographic expanse and a wide range of ecological conditions, China harbors a high diversity of *Trichoderma* species. The variation in climate, soil types, and vegetation across different regions provides favorable niches for the evolution and distribution of numerous *Trichoderma* taxa, thereby contributing to the country’s fungal biodiversity. In recent years, numerous new species of *Trichoderma* have been reported by Chinese researchers, significantly expanding the known diversity of the genus (Zhu and Zhuang 2015; Chen and Zhuang 2016; Qin and Zhuang 2016b; Sun et al. 2016; Chen and Zhuang 2017; Li et al. 2018; Qiao et al. 2018; Zheng et al. 2021; Zhang et al. 2022; Ma et al. 2024; Zhao et al. 2025).

*Trichoderma* is a species-rich and taxonomically complex genus, with substantial morphological and genetic variation among closely related taxa. Early classifications relied mainly on phenotypic traits such as conidiophore and conidial morphology and colony characters (Jaklitsch 2009), but extensive character overlap and phenotypic plasticity often limited reliable species identification. With advances in molecular systematics, multiple major clades have been recognized in *Trichoderma*, including Brevicompactum, Harzianum, Hamatum, Longibrachiatum, Semiorbis, and Viride (Qin and Zhuang 2017; Zhao et al. 2025). Recent studies commonly employ multilocus phylogenetic analyses, in which protein-coding loci such as the RNA polymerase II second largest subunit (*rpb*2) and translation elongation factor 1-alpha (*tef*1-α) provide strong resolution for species delimitation and phylogenetic placement (Jaklitsch and Voglmayr 2015; Cai and Druzhinina 2021).

Decaying wood represents a key habitat supporting high *Trichoderma* diversity in lignocellulosic environments. According to Index Fungorum, more than 250 *Trichoderma* species have been recorded from decaying wood. Between 2021 and 2025, a total of 109 new *Trichoderma* species were described, of which 76 were reported from China, further highlighting China as a major hotspot for *Trichoderma* diversity (Cai and Druzhinina 2021; Zhang et al. 2022; Zhao et al. 2025). This pattern is consistent with the country’s broad geographic range and diverse ecological conditions, which provide abundant niches for the evolution and distribution of *Trichoderma* taxa. Accordingly, systematic sampling of decaying wood across different regions of China was conducted to further assess *Trichoderma* diversity.

In recent years, numerous studies have substantially advanced the taxonomy of *Trichoderma*, for example, through comprehensive multilocus phylogenetic analyses, integrative species delimitation, and the revision of species complexes. Using multilocus DNA data from 93 global strains, Chaverri et al. (2015) uncovered a complex speciation history within the *Hypocrealixii/Trichoderma harzianum* species complex, identifying multiple genetically distinct species, several asexual lineages, and a number of early-diverging lineages. Cai and Druzhinina (2021) compiled a comprehensive taxonomic inventory of *Trichoderma* species and established standardized molecular identification guidelines using three DNA barcodes (ITS, *rpb*2, and *tef*1-α), addressing the complexity and rapid expansion of *Trichoderma* taxonomy. In this study, 209 *Trichoderma* specimens were collected from decaying wood in five provinces of China (Guizhou, Hebei, Hubei, Jilin, and Liaoning). Based on morphological examination and phylogenetic analyses of *rpb*2 and *tef*1-α sequences, 25 species were recognized, including five novel taxa described herein. These five new species span five major phylogenetic clades of *Trichoderma* and include an unexpected combination of clavate stromata and psychrophilic affinities, thereby expanding the known morphological and evolutionary diversity of the genus.

## Materials and methods

### Sample collection

Samples were collected from decaying wood in five provinces in China (Guizhou, Hebei, Hubei, Jilin, and Liaoning) during 2023–2025. Voucher specimens were dried, preserved, and deposited in the Herbarium of Mycology, Jilin Agricultural University (HMJAU).

### Isolation of strains

Pure cultures were obtained by single-ascospore isolation from fresh stromata of sexual morphs or by single-conidium isolation from asexual structures on substrates. Fresh stromata were crushed in sterile distilled water to release ascospores, whereas conidia were suspended directly in sterile distilled water. Suspensions were spread onto synthetic low-nutrient agar (SNA; Nirenberg 1976) and incubated at 25 °C overnight. Germinated ascospores or conidia were transferred to potato dextrose agar (PDA) and incubated at 25 °C. Cultures were maintained at 4 °C, and representative strains were preserved at –80 °C and deposited in the China General Microbiological Culture Collection Center (CGMCC).

### Morphological characterization and growth rate determination

Dried stromata were rehydrated, and longitudinal sections (6–10 μm) were prepared using a freezing microtome (Leica CM1950 S, Leica Biosystems Nussloch GmbH Inc., Heidelberger, Germany). Micromorphological characters were examined using a Zeiss Axioskop 2 Plus microscope (Carl Zeiss, Germany) equipped with a digital camera.

Cultures were incubated on Cornmeal Dextrose Agar (CMD), PDA, and SNA at 25 °C. Colony morphology, including color, texture, and growth rate, was recorded daily until the colony covered the Petri dish. Microscopic characters, including conidiophore branching patterns, phialide shape, and conidial morphology, were documented following standard practice (Zhu and Zhuang 2015).

### DNA extraction, PCR amplification, and sequencing

Genomic DNA was extracted from mycelium harvested from PDA cultures incubated for 1–2 weeks using the DNA Secure Plant Kit (TIANGEN; Beijing, China), following the manufacturer’s instructions. The *rpb*2 region was amplified using primer pairs fRPB2-5f/fRPB2-7cr (Liu et al. 1999) and, when appropriate, RPB-F2/RPB-R2 (Samuels et al. 2012). The *tef*1-α region was amplified using EF1-728F/TEF1LLErev (Chaverri et al. 2003; Jaklitsch et al. 2005).

PCR was performed in 25 μL reactions containing 12.5 μL 2 × Taq Plus Master Mix (Biosharp, Anhui, China), 1 μL of each primer (10 μM), 3 μL of genomic DNA, and 7.5 μL ddH_2_O. PCR was performed for 35 cycles, with an annealing temperature of 57 °C for *rpb*2 and 55 °C for *tef*1-α. Purified PCR products were sequenced using an ABI 3730XL DNA Sequencer at Beijing Tianyihuiyuan Bioscience and Technology Co., Ltd. (Beijing, China).

### Phylogenetic analysis

Consensus sequences were assembled from forward and reverse reads and manually edited in BioEdit v.7.0.5.3 (Hall 2012). Preliminary identification was assessed by BLAST searches against the NCBI nucleotide database, and closely related taxa were selected for phylogenetic analyses (Table 1). Alignments for *rpb*2 and *tef*1-α were generated using ClustalW v.2.1 and then manually inspected and trimmed to comparable regions following the DNA barcoding loci recommended for *Trichoderma* identification (Cai and Druzhinina 2021). Because *tef*1-α contains highly variable regions and length polymorphisms in some taxa, a subset of published *tef*1-α sequences could not be aligned reliably across the full sampling under strict criteria and was therefore excluded from the *tef*1-α dataset.

**Table 1.** Strain numbers and corresponding GenBank accession numbers of sequences used for phylogenetic analyses. “–” indicates that sequence data are not available. The sequences newly generated in this study are highlighted in bold. T = ex-type strain.

Species	Type	Strain	GenBank accession numbers	
			*rpb2*	*tef1*-α
*Trichoderma americanum* Jaklitsch & Voglmayr	T	GJS 92-93	DQ835455	–
*T. anaharzianum* Z.F. Yu & X. Du	T	YMF 1.00383	MH158995	MH183182
*T. angustum* W.T. Qin & W.Y. Zhuang	T	HMAS 273784	KX026967	KX026959
*T. applanatum* Z.X. Zhu & W.Y. Zhuang	T	CGMCC 3.17526	KJ634726	KJ634759
*T. arundinaceum* Zafari, Gräfenhan & Samuels	T	CBS 119575	EU338326	EU338291
*T. arundinaceum*		GJS 05-183	EU338302	EU338274
*T. asiaticum* Z.F. Yu & X. Du	T	YMF 1.00352	MH158994	MH183183
*T. asperellum* Samuels, Lieckf. & Nirenberg	T	CBS 433.97	EU248617	AY376058
*T. atroviride* P. Karst	T	CBS 142.95	EU341801	AF456891
*T. auranteffusum* Jaklitsch	T	CBS 119284	FJ860520	FJ860613
*T. austriacum* Jaklitsch	T	CBS 122494	FJ860525	FJ860619
*T. balearicum* Jaklitsch & Voglmayr	T	CBS 133222	KJ665242	KJ665434
*T. barbatum* Jaklitsch & Voglmayr	T	GJS 04-308	HQ342286	HQ342223
*T. brevicompactum* G.F. Kraus, C.P. Kubicek & W. Gams		GJS 04-381	EU338317	EU338299
*T. brevicompactum*		CBS 112443	EU338319	EU338281
*T. britdaniae* Jaklitsch & Voglmayr	T	K 89878	JQ685881	JQ685865
*T. britdaniae*		WU 31610	JQ685880	JQ685866
*T. caesareum* Samuels	T	GJS 01-225	HQ342279	HQ342216
*T. calamagrostidis* Jaklitsch	T	CBS 121133	FJ860528	FJ860622
*T. catoptron* P. Chaverri & Samuels	T	GJS 02-76	AY391900	–
*T. ceciliae* Jaklitsch & Voglmayr	T	CBS 130010	KJ665247	KJ665444
*T. ceramicum* P. Chaverri & Samuels	T	CBS 114576	FJ860531	FJ860628
*T. ceramicum*		S353	KJ665248	KJ665445
*T. changbaiense* K. Chen & W.Y. Zhuang	T	HMAS 247198	MF371209	MF371224
*T. citrinoviride* Bissett	T	DAOM 172792	KJ842210	KJ713208
*T. citrinum* (Pers.) Jaklitsch, W. Gams & Voglmayr	T	C.P.K. 960	FJ179603	FJ860631
*T. clavaticapitatum* Z.Q. Zeng, X.H. Wang & W.Y. Zhuang	T	HKAS 131918	OR965857	–
*T. confluens* W.T. Qin & W.Y. Zhuang	T	HMAS 244993	KT001964	KT001959
*T. crystalligenum* Jaklitsch	T	CBS 118980	DQ345347	DQ345342
*T. crystalligenum*		C.P.K.1604	–	DQ345343
*T. crystalligenum*		C.P.K.1911	DQ345348	–
*T. decipiens* Jaklitsch & Voglmayr	T	CBS 121307	–	FJ860635
*T. decipiens*		GJS 91-101	DQ835520	–
*T. deliquescens* (Sopp) Jaklitsch	T	CBS 121131	FJ179609	OQ471215
*T. estonicum* P. Chaverri & Samuels	T	GJS 96-129	AF545514	–
*T. estonicum*		CBS 121556	FJ860536	FJ860637
*T. eucorticioides* (Overton) Jaklitsch & Voglmayr	T	GJS 99-61	DQ835518	DQ835502
*T. fassatiae* A. Nováková, Kubátová, Valinová, Hubka & M. Kolařík	T	CBS 140105	LN866276	LN866277
*T. fertile* Bissett	T	DAOM 167161	AF545546	KJ871131
*T. flavobritdaniae*	T	HMJAU 34748 = CGMCC 7.669	PX024840	PX024844
*T. floccosum* Samuels	T	GJS 01-238	HQ342281	HQ342218
*T. foliatum*	T	HMJAU 34755 = CGMCC 7.672	PX665987	PX682156
*T. fomiticola* Jaklitsch	T	CBS 121136	FJ860538	FJ860639
*T. fomiticola*		C.P.K. 3137	FJ860539	FJ860640
*T. fructicola* Y.B. Zhang & W.Y. Zhuang	T	HMAS 275663	MG383484	MG383490
*T. fulviclavum*	T	HMJAU 34754 = CGMCC 7.673	PX665986	PX682158
*T. fulviclavum*		HMJAU 34758	PX665985	PX682157
*T. grande* W.T. Qin & W.Y. Zhuang	T	HMAS 248749	KX066266	KX066254
*T. guizhouense* Q.R. Li, McKenzie & Yong Wang bis	T	CBS 131803	JQ901400	JN215484
*T. hamatum* (Bonord.) Bainier	T	DAOM 167057	AF545548	AF456911
*T. harzianum* Rifai	T	CBS 226.95	AF545549	AF534621
*T. hausknechtii* Jaklitsch & Voglmayr	T	CBS 133493	KJ665276	KJ665515
*T. hebeiense* K. Chen & W.Y. Zhuang	T	HMAS 248743	KX344439	KX344434
*T. helicolixii* Jaklitsch & Voglmayr	T	CBS 133499	KJ665278	KJ665517
*T. hunua* (Dingley) Jaklitsch & Voglmayr	T	CBS 238.63	KJ665279	KJ665519
*T. ivoriense* Samuels	T	GJS 01-312	HQ342280	HQ342217
*T. koningiopsis* Samuels, C. Suárez & H.C. Evans	T	GJS 93-20	EU241506	DQ284966
*T. lanuginosum* Samuels	T	GJS 01-176	HQ342283	HQ342221
*T. lanuginosum*		GJS 01-174	HQ342284	HQ342220
*T. leucopus* Jaklitsch	T	CBS 122499	FJ179605	FJ179571
*T. liaoningense*	T	HMJAU 34752 = CGMCC 7.671	PX024842	PX024847
*T. limonium* W.T. Qin & W.Y. Zhuang	T	HMAS 248751	KX066259	KX066247
*T. limonium*		HMAS 248754	KX066260	KX066248
*T. lixii* (Pat.) P. Chaverri	T	CBS 110080	KJ665290	FJ716622
*T. longibrachiatum* Rifai	T	ATCC 18648	HQ260615	AY937412
*T. longipile* Bissett	T	CBS 120953	FJ860542	FJ860643
*T. luteocrystallinum* Jaklitsch	T	CBS 123828	FJ860544	FJ860646
*T. margaretense* Jaklitsch	T	C.P.K. 3127	FJ860529	FJ860625
*T. medusae* Samuels	T	GJS 01-171	HQ342277	HQ342214
*T. medusae*		GJS 01-166	HQ342278	HQ342215
*T. megalocitrinum* (Yoshim. Doi) Jaklitsch & Voglmayr	T	BEO 0009	AF545563	–
*T. melanomagnum* P. Chaverri & Samuels	T	GJS 99-153	AY391926	–
*T. microcitrinum* (Yoshim. Doi) Jaklitsch & Voglmayr	T	GJS 97-248	DQ835462	–
*T. mienum* C.S. Kim, Nakagiri & N. Maek.	T	TUFC 61533	AB856754	AB856681
*T. moravicum* Jaklitsch	T	CBS 120539	–	FJ860651
*T. moravicum*		C.P.K. 2489	FJ860549	–
*T. notatum* Z.J. Cao & W.T. Qin	T	JZBQT1Z5	OP832381	OP832396
*T. nybergianum* (T. Ulvinen & H.L. Chamb.) Jaklitsch & Voglmayr	T	CBS 122500	FJ179611	FJ179575
*T. oblongisporum* Bissett	T	DAOM 176226	KJ842199	KJ871142
*T. oligosporum* Z.X. Zhu & W.Y. Zhuang	T	HMAS 252870	KJ634731	KJ634764
*T. orientale* (Samuels & Petrini) Jaklitsch & Samuels		S187	JQ685884	JQ685868
*T. orientale*	T	GJS 88-81	–	EU401581
*T. parareesei* Jaklitsch & Atanasova	T	CBS 125925	HM182963	GQ354353
*T. parestonicum* Jaklitsch		CBS 120636	FJ860565	FJ860667
*T. parestonicum*		C.P.K. 2427	FJ860564	FJ860666
*T. parvostromatum*	T	HMJAU 34749 = CGMCC 7.670	PX024843	PX024845
*T. phellinicola* Jaklitsch	T	CBS 119283	FJ860569	–
*T. polysporum* (Link) Rifai	T	CBS 112265	KJ665349	–
*T. protopulvinatum* (Yoshim. Doi) Jaklitsch & Voglmayr	T	CBS 739.83	DQ835463	FJ860679
*T. protrudens* Samuels & Chaverri	T	DIS 119F	EU338322	EU338289
*T. pseudobritdaniae* W.T. Qin & W.Y. Zhuang	T	HMAS 271355	KT224466	KT224462
*T. pseudogelatinosum* (M. Komatsu & Yoshim. Doi) C.S. Kim	T	CNUN 309	HM920173	–
*T. pseudolacteum* C.S. Kim & N. Maek.	T	TUFC 61490	–	JX238493
*T. psychrophilum* Jaklitsch		Hy8	JN133564	JN133574
*T. psychrophilum*		C.P.K. 1602	FJ860575	FJ860680
*T. pulvinatum* (Fuckel) Jaklitsch & Voglmayr	T	CBS 121279	FJ860577	FJ860683
*T. reesei* E.G. Simmons	T	DAOM 167654	KJ842213	KJ713193
*T. rhododendri* Jaklitsch & Voglmayr	T	CBS 119288	FJ860578	FJ860685
*T. rodmanii* (Samuels & P. Chaverri) Jaklitsch & Voglmayr	T	GJS 91-88	EU338324	EU338286
*T. rossicum* Bissett, C.P. Kubicek & Szakacs	T	DAOM 230011	KJ842191	AY937441
*T. saturnisporopsis* Samuels & Jaklitsch	T	S19	JQ685885	JQ685869
*T. saturnisporopsis*		RSI	PV157957	PV157965
*T. semiorbis* (Berk.) Jaklitsch & Voglmayr	T	GJS 99-108	JN133567	JN133576
*T. semiorbis*		DAOM 167636	AF545522	AF545568
*T. seppoi* Jaklitsch	T	CBS 122498	FJ179617	FJ179581
*T. shennongjianum*. K. Chen & W.Y. Zhuang	T	HMAS 245009	KT735259	KT735253
*T. sichuanense* K. Chen & W.Y. Zhuang	T	HMAS 248737	KX344437	KX344428
*T. simile* Z.F. Yu & Y.F. Lv	T	YMF 1.06201	MT052184	MT070154
*T. simmonsii* P. Chaverri, F.B. Rocha, Samuels, Degenkolb & Jaklitsch	T	GJS 91-138	FJ442757	AF443935
*T. stercorarium* (Barrasa, A.T. Martínez & G. Moreno) Jaklitsch & Voglmayr	T	CBS 148.85	EF469103	FJ860607
*T. strictipile* Bissett	T	C.P.K. 1601	FJ860594	FJ860704
*T. stromaticum* Samuels & Pardo-Schulth	T	GJS 97-183	HQ342245	AY937418
*T. stromaticum*		GJS 97-180	HQ342226	HQ342166
*T. subsulphureum* (Syd. & P. Syd.) Jaklitsch & Voglmayr		M 141	DQ835522	–
*T. sulphureum* (Schwein.) Jaklitsch & Voglmayr	T	CBS 119929	FJ179620	FJ860710
*T. tiantangzhaiense* Z.X. Zhu & W.Y. Zhuang	T	HMAS 252872	KJ634730	–
*T. tibetense* K. Chen & W.Y. Zhuang	T	HMAS 245010	KT735261	KT735254
*T. tremelloides* Jaklitsch	T	CBS 121140	FJ860603	FJ860714
*T. tropicosinense* P.G. Liu, Z.X. Zhu & W.Y. Zhuang	T	HMAS 252546	KF923313	KF923286
*T. turrialbense* Samuels, Degenkolb, K.F. Nielsen & Gräfenhan	T	CBS 112445	EU338321	EU338284
*T. turrialbense*		BBA 72294	EU338320	EU338282
*T. vermipilum* Samuels	T	PPRI 3559	HQ342282	HQ342219
*T. vermipilum*		TXM 15	KJ801863	KJ652480
*T. verticillatum* K. Chen & W.Y. Zhuang	T	HMAS 248740	KX344438	KX344431
*T. victoriense* (Overton) Jaklitsch & Voglmayr	T	CBS 140064	DQ835517	–
*T. virens* (J.H. Mill., Giddens & A.A. Foster) Arx		DIS 162	FJ442696	–
*T. virens*	T	CBS 249.59	–	EU280047
*T. viride* Pers.	T	CBS 119325	EU711362	–
*T. zeloharzianum* Z.F. Yu & X. Du	T	YMF 1.00268	MH158996	MH183181
*Protocrea pallida* (Ellis & Everh.) Jaklitsch, K. Põldmaa & Samuels	T	CBS 299.78	EU703947	EU703900

Phylogenetic analyses were conducted separately for the *rpb*2 and *tef*1-α datasets and for the combined *rpb*2 and *tef*1-α datasets using Maximum Likelihood (ML) and Bayesian Inference (BI). The best-fit nucleotide substitution model for each dataset was selected using MrModeltest 2 v.2.4 (Nylander 2004), and all datasets supported GTR+I+G as the optimal model. The ML analyses were performed in IQ-TREE v.2.4.0 (Nguyen et al. 2015). BI analyses were conducted in MrBayes v.3.2.7 (Ronquist et al. 2012) with Markov chain Monte Carlo (MCMC) runs of 2,000,000 generations, sampling every 100 generations; the first 25% of trees were discarded as burn-in. Support values were assessed using ML bootstrap proportions (MLBP) and Bayesian posterior probabilities (BIPP), with MLBP ≥ 75% and BIPP ≥ 0.90 indicated on the trees. Trees were visualized in FigTree v.1.4.3. Strain information and GenBank accession numbers used for phylogenetic analyses are provided in Table 1.

## Results

### Phylogenetic analysis

**Figure 1.**
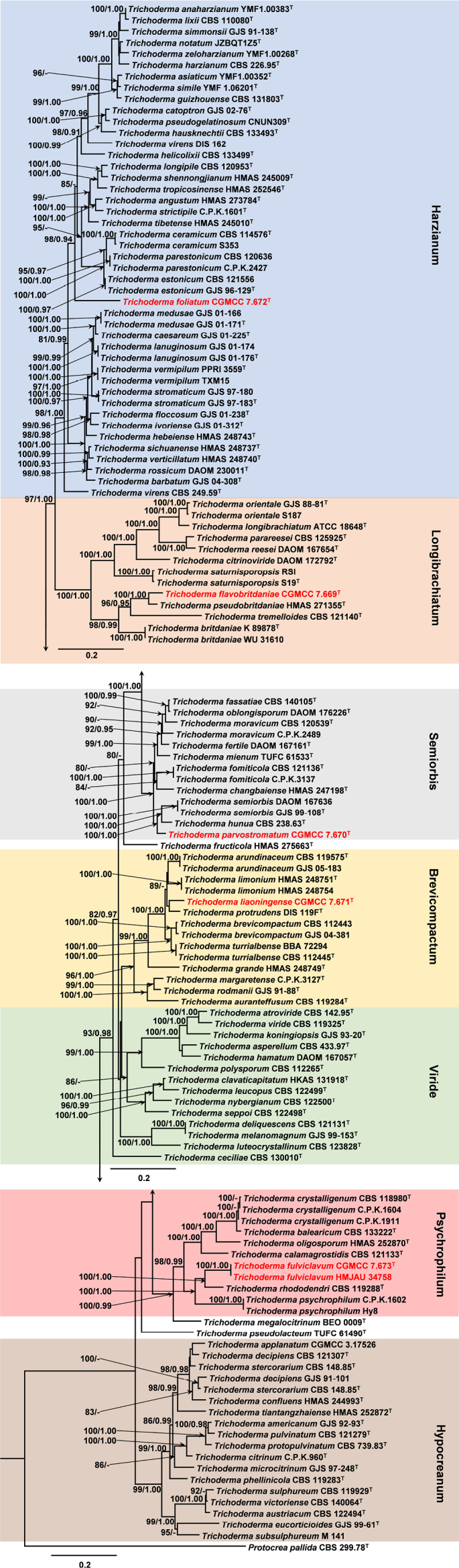
Phylogenetic tree based on the maximum likelihood analysis of the combined *rpb*2 and *tef*1 dataset. MLBP above 75% (left) and BIPP above 0.90 (right) are indicated at the nodes. New species proposed here are indicated in red. ^T^ = ex-type strain.

In total, 128 strains representing 111 *Trichoderma* species were included in the phylogenetic analyses, with *Protocrea pallida* selected as the outgroup taxon. ML and BI analyses yielded largely congruent topologies for both loci. A combined phylogeny based on *rpb*2 and *tef*1-α was therefore used to present the main results (Fig. 1), with MLBP ≥ 75% and BIPP ≥ 0.90 indicated at the nodes. In the combined tree, taxa were resolved into seven major clades: Harzianum, Longibrachiatum, Semiorbis, Brevicompactum, Viride, Hypocreanum, and Psychrophilum. Most major clades received strong Bayesian support. The phylogenetic trees inferred from the individual *rpb*2 and *tef*1-α datasets are provided in the Suppl. material 1: figs S1, S2. These single-locus trees showed topologies largely consistent with the combined phylogeny, differing mainly in support values at some nodes.

The five novel species formed distinct lineages and were assigned to five different clades. *Trichoderma flavobritdaniae* was placed in the Longibrachiatum clade and was resolved as sister to *T. pseudobritdaniae* (HMAS 271355), with strong support (MLBP/BIPP = 100/1.00). *Trichoderma foliatum* constituted an independent lineage within the Harzianum clade, with strong support (98/0.94). *Trichoderma parvostromatum* clustered within the Semiorbis clade and was closely related to *T. hunua* (CBS 238.63) and *T. semiorbis*, with strong support (100/1.00). *Trichoderma liaoningense* was positioned in the Brevicompactum clade and appeared most closely related to *T. protrudens* (DIS 119F), although this relationship received weak support. *Trichoderma fulviclavum* was nested within the Psychrophilum clade and was sister to *T. rhododendri* (CBS 119288) and *T. psychrophilum*, with strong support (100/1.00).

### Taxonomy

#### Trichoderma flavobritdaniae

Taxon classificationFungiHypocrealesHypocreaceae

G.S. Jin & Z.X. Zhu sp. nov.

D093DAA6-8334-5942-9C6B-8EBA9570667E

859956

Figs 2, 3

##### Etymology.

From *flavus* (yellow) + *britdaniae*, referring to the yellow pigment produced on PDA and the overall resemblance of the sexual morph to *T. britdaniae*.

##### Diagnosis.

Stromata solitary to gregarious, pulvinate, light brown to brown, turning dark purple in 3% KOH; colonies on PDA producing a conspicuous bright yellow pigment; conidiophores verticillium-like with comparatively long phialides.

##### Typification.

China • Guizhou Province, Duyun City, Qingyunhu National Forest Park, 26°15'3"N, 107°31'12"E, 853 m alt., on wood and fungi, 18 Aug. 2023, Z.X. Zhu 1545G (Holotype HMJAU 34748, ex-type culture CGMCC 7.669).

##### Sexual morph.

***Stromata*** pulvinate solitary, scattered or aggregated in small groups, centrally attached with margins free, 1.2–1.5 mm diam, outline circular to irregular. Stromata white and hairy when young, becoming brown at maturity; turning dark purple in 3% KOH. Stromatal surface velvety when young, becoming plane at maturity, slightly wrinkled; perithecial protuberances inconspicuous, ostioles visible as black or brown dots.

**Figure 2.**
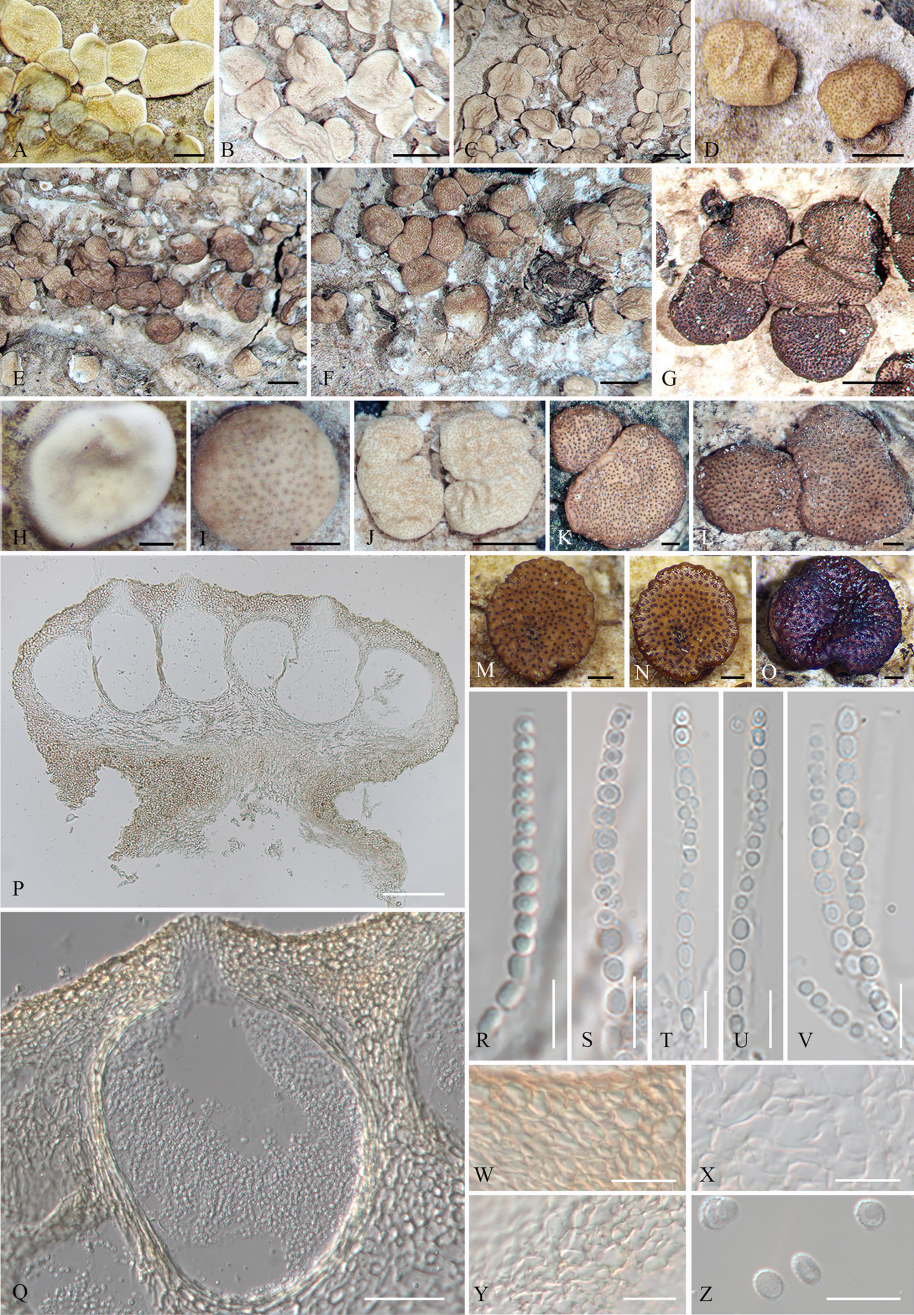
Sexual morph of *Trichoderma flavobritdaniae* (HMJAU 34748). **A–M**. Dry mature stromata; **N**. Mature stromata after reconstitution in water; **O**. Mature stromata in 3% KOH after reconstitution in water; **P**. Stromata in section; **Q**. Perithecium in section; **R–V**. Asci with ascospores; **W**. Cortical and subcortical tissue in section; **X**. Subperithecial tissue in section; **Y**. Basal tissue of stroma in section; **Z**. Ascospores. Scale bars: 1 mm (**A–G, J**); 0.25 mm (**H, I, K–O**); 100 µm (**P**); 20 µm (**Q, W–Y**); 10 µm (**R–V, Z**).

In section stroma, ***perithecia*** flask-shaped, elliptical or globose, (156–)172–225(–260) × (91–)110–133(–140) µm (*n* = 20). ***Ostioles*** (25–)38–43(–59) µm long (*n* = 20), plane or projecting up to 16 µm, 18–23 µm wide at the apex (*n* = 20). ***Peridium*** brown, (6.3–)7.4–8.2(–9.1) µm thick at base and sides (*n* = 20). Cortical tissue of ***textura angularis***, cells hyaline, thick-walled, (4.8–)6.7–8(–9.6) × (3.4–)3.8–7.1(–7.3) μm (*n* = 20); subcortical tissue of ***textura angularis*** mixed with ***textura intricata***, cells hyaline, thin-walled, (4.3–)5–6.8(–8.7) × (2.3–)3.1–4.4(–5.3) μm (*n* = 20); hyphae hyaline, thin-walled, 2–4.3 μm wide (*n* = 20); subperithecial tissue of ***textura angularis*** mixed with ***textura epidermoidea***, cells hyaline, thin-walled, (6.6–)7.4–16.8(–18.2) × (5.7–)6.6–15.1(–17.9) μm (*n* = 20); tissue at the base of ***textura angularis***, cells hyaline, thin-walled, (6.8–)7.5–14.4(–16) × (4.1–)4.5–6.9(–7.5) μm (*n* = 20). ***Asci*** cylindrical, (55–)56–60(–64) × (3.6–)3.8–4(–4.2) µm (*n* = 20). ***Ascospores*** dimorphic, hyaline, verrucose or spinulose with tubercles, distal cells subglobose to globose (2.6–)2.9–4.4(–4.8) × (2.3–)2.6–3.7(–3.9) µm (*n* = 20), l/w 1–1.2 (–1.4), proximal cells oblong or globose (2.8–)3.5–4.4(–4.7) × 2.4–2.8(–3.4) µm (*n* = 20), l/w 1.1–1.9.

**Figure 3.**
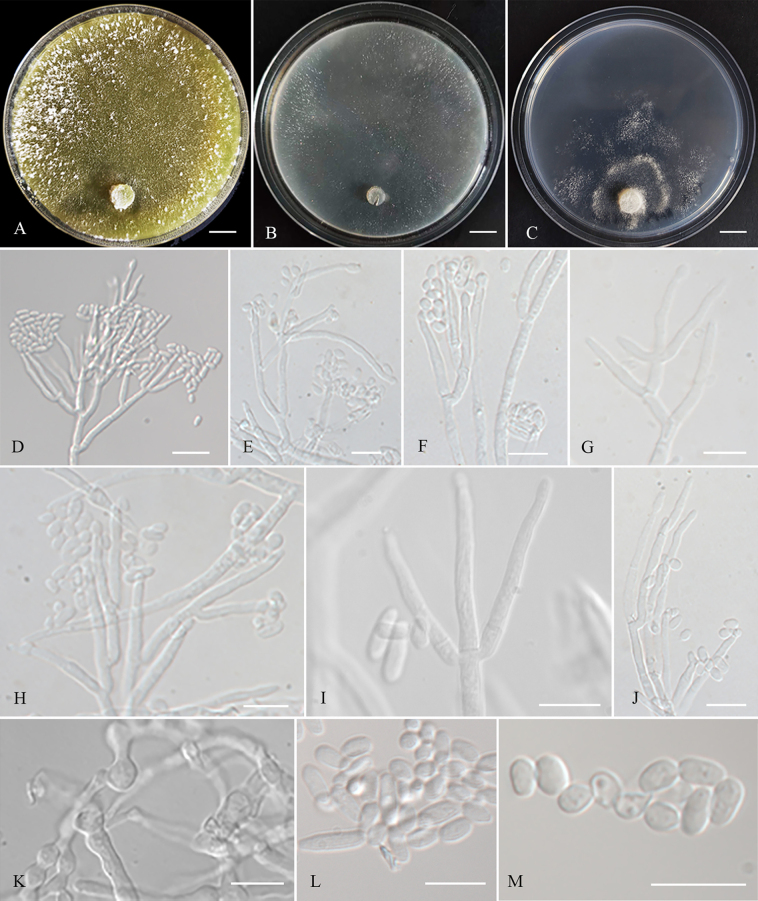
Colonies and microscopic characteristics of *Trichoderma flavobritdaniae* (Holotype CGMCC 7.669). **A–C**. Colonies after 3 weeks at 25 °C on media (**A**PDA, **B**CMD, **C**SNA); **D–J**. Conidiophores and phialides; **K**. Chlamydospore; **L, M**. Conidia. Scale bars: 1 cm (**A–C**); 20 μm (**D**); 10 μm (**E–M**).

##### Cultures and asexual morph.

On PDA at 25 °C, colony radius 33–37 mm after 72 h, with the plate fully covered after 7 days. Colonies white, with sparse aerial hyphae. Conidiation rare, appearing after 7 days; pustules white, sparsely distributed near the colony margin. ***Conidiophores*** loosely disposed, verticillium-like, arising in whorls of 2–4 phialides or with solitary phialides along the main axis. ***Phialides*** straight, lageniform to subulate, (13–)15.5–28.4(–33.8) × (2.4–)2.6–3.9(–4.3) μm (*n* = 20), l/w (4.5–)5.5–10(–11.6), (1.6–)1.9–3(–3.4) μm wide at the base (*n* = 20). ***Conidia*** hyaline, ellipsoidal to oblong, or subglobose to globose, smooth, (5–)5.4–8(–9.2) × (2.6–)2.8–3.5(–3.7) μm (*n* = 20), l/w (1.4–)1.5–3.1(–3.5). ***Chlamydospores*** present, terminal and intercalary, globose or ellipsoid, (6.3–)6.5–7.7(–8.4) × (4.9–)5.4–6.7(–8.6) μm (*n* = 20), l/w 1.1–1.3. Odor absent; bright yellow pigment present.

On CMD at 25 °C, colony radius 25–32 mm after 72 h, with the plate fully covered after 7 days. Colonies white, with sparse aerial hyphae. Conidiation appearing after 15 days; pustules white, rare, and sparsely disposed. Chlamydospores numerous, globose or ellipsoid. Odor absent; pale yellow pigment present.

On SNA at 25 °C, colony radius 2–7 mm after 72 h, with the plate fully covered after 30 days. Colonies white, irregular outline, with sparse aerial hyphae. Conidiation appearing after 15 days; pustules zonate, spreading scattered, generate one irregular circle. Odor absent; no pigment observed.

##### Notes.

*Trichoderma flavobritdaniae* is morphologically similar to *T. pseudobritdaniae* and *T. britdaniae*, sharing a generally similar stromatal coloration. However, unlike *T. britdaniae*, both *T. flavobritdaniae* and *T. pseudobritdaniae* produce pulvinate stromata that are markedly smaller in size (up to 100 mm in *T. britdaniae*; 2–3.5(–5) mm in *T. pseudobritdaniae*; 1.2–1.5 mm in *T. flavobritdaniae*) (Jaklitsch and Voglmayr 2012; Qin and Zhuang 2016a). The perithecia of the three species are comparable in size, whereas the asci of *T. flavobritdaniae* [(55–)56–60(–64) μm] are shorter than those of *T. britdaniae* [(52–)60–75(–86) μm] and *T. pseudobritdaniae* [(62–)68–82(–89) μm]. In the asexual morph, *T. flavobritdaniae* is readily distinguished by the production of a conspicuous bright yellow pigment on PDA, a feature absent in *T. pseudobritdaniae*. In addition, *T. flavobritdaniae* possesses longer phialides [(13–)15.5–28.4(–33.8) μm vs. (7–)8.5–18(–20) μm] and longer conidia [(5–)5.4–8(–9.2) μm vs. (2.5–)3–7(–7.5) μm] than *T. pseudobritdaniae* (Jaklitsch and Voglmayr 2012; Qin and Zhuang 2016a). Detailed morphological differences among the three species are summarized in Table 2.

**Table 2.** Morphological comparisons of *Trichoderma flavobritdaniae* with *T. britdaniae* and *T. pseudobritdaniae*. Characters showing diagnostic differences are highlighted with gray shading. A dash (–) indicates that the character is not reported.

Character		*T. britdaniae* (Jaklitsch and Voglmayr 2012)	*T. pseudobritdaniae* (Qin and Zhuang 2016a)	*T. flavobritdaniae* (this study)
Stromata	Color	Light to medium brown, sometimes reddish brown	Brownish yellow or grayish yellow	white when young, becoming brown at maturity
	Color in 3% KOH	Orange–red, finally brown	Brownish red	Dark purple
	Shape	Scattered or undulate	Pulvinate	Pulvinate
	Diameter (mm)	up to 100	2–3.5(–5)	1.2–1.5
	Thickness (mm)	up to 3	0.7–1.0	0.15–0.23
Ostioles	Color	Dark brown or reddish brown with light center	Dark brown or gray black	Black
	Length (μm)	(46–)54–68(–73)	(53–)55–66	(25–)38–43(–59)
	Width at the apex (μm)	(11–)15–32(–43)	26–37(–40)	18–23
Perithecia	Color of peridium	Yellow	Pale yellow brown	Pale yellow brown
	Length (μm)	(174–)200–235(–255)	(195–)224–263(–283)	(156–)172–225(–260)
	Width (μm)	(60–)80–125(–165)	(111–)119–142(–171)	(91–)110–133(–140)
	Asci length (μm)	(52–)60–75(–86)	(62–)68–82(–89)	(55–)56–60(–64)
	Asci width (μm)	(3.7–)4–5(–5.5)	4.2–5(–5.5)	(3.6–)3.8–4(–4.2)
Ascospore	Shape and ornamentation	Smooth to finely spinulose, cells subglobose to globose	Spinulose or verruculose, cells globose, subglobose, or broad-ellipsoidal	Spinulose with tubercles, cells subglobose to globose or oblong to globose
	Color	Hyaline	Hyaline	Hyaline
	Distal length (μm)	(2.0–)2.5–3.0(–3.8)	2.8–3.5	(2.6–)2.9–4.4(–4.8)
	Distal width (μm)	(2.0–)2.5–3.0(–3.5)	(2.2–)2.5–3.2	(2.3–)2.6–3.7(–3.9)
	Distal l/w ratio	0.9–1.1(–1.4)	1.0–1.5	1–1.2(–1.4)
	Proximal length (μm)	(2.0–)2.5–3.0(–3.8)	(2.4–)2.9–3.7(–4.2)	(2.8–)3.5–4.4(–4.7)
	Proximal width (μm)	(2.0–)2.5–3.0(–3.5)	2.1–3.2	2.4–2.8(–3.4)
	Proximal l/w ratio	0.9–1.1(–1.4)	1.0–1.5(–1.9)	1.1–1.9
Colony radius after 72 h at 25 °C (mm)	CMD	–	15–18	25–32
	PDA	–	3–5	33–37
	SNA	–	13–15	2–7
Phialides	Length (μm)	–	(7–)8.5–18(–20)	(13–)15.5–28.4(–33.8)
	Width (μm)	–	(2.5–)2.8–4(–4.5)	(2.4–)2.6–3.9(–4.3)
	l/w ratio	–	(2.1–)2.7–4.7(–7.2)	(4.5–)5.5–10(–11.6)
	Basal width (μm)	–	1.3–3.3(–3.5)	(1.6–)1.9–3(–3.4)
Conidia	Shape		Ellipsoidal or oblong, also subglobose or oval, sometimes cylindrical, smooth	Ellipsoidal to oblong, or subglobose to globose, smooth
	Color	–	Hyaline	Hyaline
	Length (μm)	–	(2.5–)3–7(–7.5)	(5–)5.4–8(–9.2)
	Width (μm)	–	(2.5–)3–4.4(–4.5)	(2.6–)2.8–3.5(–3.7)
	l/w ratio	–	1.0–1.9(–2.1)	(1.4–)1.5–3.1(–3.5)
Chlamydospores	Length (μm)	–	(4–)5–12	(6.3–)6.5–7.7(–8.4)
	Width (μm)	–	4–10	(4.9–)5.4–6.7(–8.6)
	l/w ratio	–	1.0–1.3	1.1–1.3
Odor and pigment		–	Absent	Odor absent; bright yellow pigment present

Phylogenetically, *T. flavobritdaniae* is placed within the Longibrachiatum clade and forms a sister relationship with *T. pseudobritdaniae* (HMAS 271355). Sequence similarities between the two species are 97.71% for *rpb*2 and 94.26% for *tef*1-α, supporting their recognition as closely related but distinct species.

#### Trichoderma foliatum

Taxon classificationFungiHypocrealesHypocreaceae

G.S. Jin & Z.X. Zhu sp. nov.

E178A9D1-EFEF-5DDE-851A-B0BF9B86B588

861392

Fig. 4

##### Etymology.

From Latin *foliatus* (= lobed, leaf-like), referring to the irregularly lobed colony margin on PDA.

##### Diagnosis.

Slow-growing on PDA, colonies with irregularly lobed margins; green pustules produced after 3–4 days. On CMD, colonies forming a loose concentric annular pattern.

##### Typification.

China • Jilin Province, Changchun City, Shuangyang District, 43°21'5"N, 125°53'43"E, 337 m alt., on rotten wood, 31 Jul. 2025, Z.X. Zhu, G.S. Jin, B. Liang, M.Y. Zhu, G.L. Zhao 2181 (Holotype HMJAU 34755, ex-type culture CGMCC 7.672).

**Figure 4.**
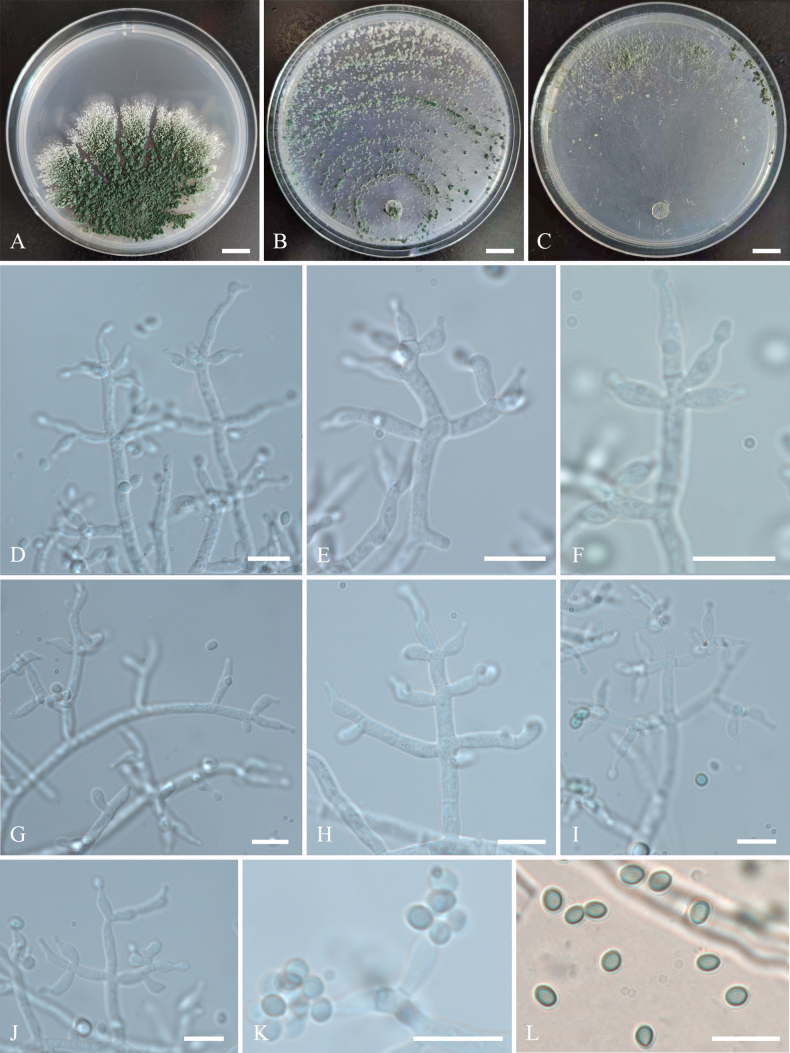
Colonies and microscopic characteristics of *Trichoderma foliatum* (Holotype CGMCC 7.672). **A–C**. Colonies at 25 °C on media (**A**PDA 21 d, **B**CMD 15 d, **C**SNA 25 d); **D–K**. Conidiophores and phialides; **L**. Conidia. Scale bars: 1 cm (**A–C**); 10 μm (**D–L**).

##### Sexual morph.

Unknown.

##### Cultures and asexual morph.

On PDA at 25 °C, colony radius 6–8 mm after 72 h, reaching 42–44 mm after 3 weeks. Colonies white, often divided into several fissured segments, margin initially circular and becoming dentate or lobed with growth, with fertile aerial hyphae. Conidiation appearing after 3–4 days; pustules contiguous, initially pale white, becoming dark green with age. ***Conidiophores*** trichoderma-like, arising in whorls of 2–5 phialides or with solitary phialides along the main axis. ***Phialides*** straight, lageniform to subulate, (5.1–)6–11.4(–12.7) × (2.2–)2.4–3.4(–3.7) μm (*n* = 30), l/w (1.5–)2.1–4.2(–4.7), 1.4–2.7 μm wide at the base (*n* = 30). ***Conidia*** green, subglobose to globose or ellipsoidal, (2.2–)2.6–4 × 2.1–3.3(–3.7) μm (*n* = 30), l/w (0.7–)1–1.4(–1.7). Odor absent; no pigment observed.

On CMD at 25 °C, colony radius 12–14 mm after 72 h, with the plate fully covered after 15 days. Colonies white, with rare aerial hyphae. Conidiation appearing after 15 days; pustules green, disposed in a loose concentric annular pattern. Odor absent; no pigment observed.

On SNA at 25 °C, colony radius 6–8 mm after 72 h, with the plate fully covered after 25 days. Colonies white, with rare aerial hyphae. Conidiation appearing after 7 days; pustules loosely scattered along the inner margin of the colony. Chlamydospores absent. Odor absent; no pigment observed.

##### Notes.

*Trichoderma foliatum* produces colonies with irregularly lobed margins on PDA, resembling those of *T. aureoviride* and *T. estonicum*. However, *T. foliatum* exhibits a markedly slower growth rate (6–8 mm after 72 h), similar to *T. aureoviride* (4–5 mm after 72 h) but clearly distinct from *T. estonicum* (29–31 mm after 72 h). The combination of slow vegetative growth and early, well-developed pustule formation represents an uncommon character state within *Trichoderma* (Jaklitsch 2009).

Phylogenetically, *T. foliatum* is resolved as a distinct lineage within the Harzianum clade. It differs from its closest GenBank BLAST result, *T. ceramicum* (CBS 114576), by 66 nucleotide substitutions in the *rpb*2 locus (969 bp), corresponding to an overall sequence similarity of approximately 93%, supporting its recognition as a distinct species.

#### Trichoderma fulviclavum

Taxon classificationFungiHypocrealesHypocreaceae

G.S. Jin & Z.X. Zhu sp. nov.

D274DAA3-F64D-57B7-A39F-389629BDC26B

861391

Figs 5, 6

##### Etymology.

The epithet *fulviclavum* refers to the fulvous (yellow-brown) clavate stromata produced by this species.

##### Diagnosis.

Stromata clavate, brown to light brown, without color change in 3% KOH; ascospores hyaline, monomorphic; asexual morph extremely slow-growing, producing brown pigment and chlamydospores, but lacking conidiation.

##### Typification.

China • Jilin Province, Changchun City, Shuangyang District, 43°21'3"N, 125°53'42"E, 356 m alt., on pine branches, 31 Jul. 2025, Z.X. Zhu, G.S. Jin, B. Liang, M.Y. Zhu, G.L. Zhao 2191 (Holotype HMJAU 34754, ex-type culture CGMCC 7.673).

##### Sexual morph.

Fresh stromata are similar to dry stromata in morphology. ***Dry Stromata*** cylindric or flattened clavate, often aggregate and grow into palmate bunches. Length up to 57 mm, 12–15 mm diam. White and hairy when young, turning light brown when mature, not changing color in KOH. Stroma surface young with fur, turns plane when mature, smooth or slightly wrinkled, perithecial protuberances not evident, ostioles appearing as black or brown dots.

**Figure 5.**
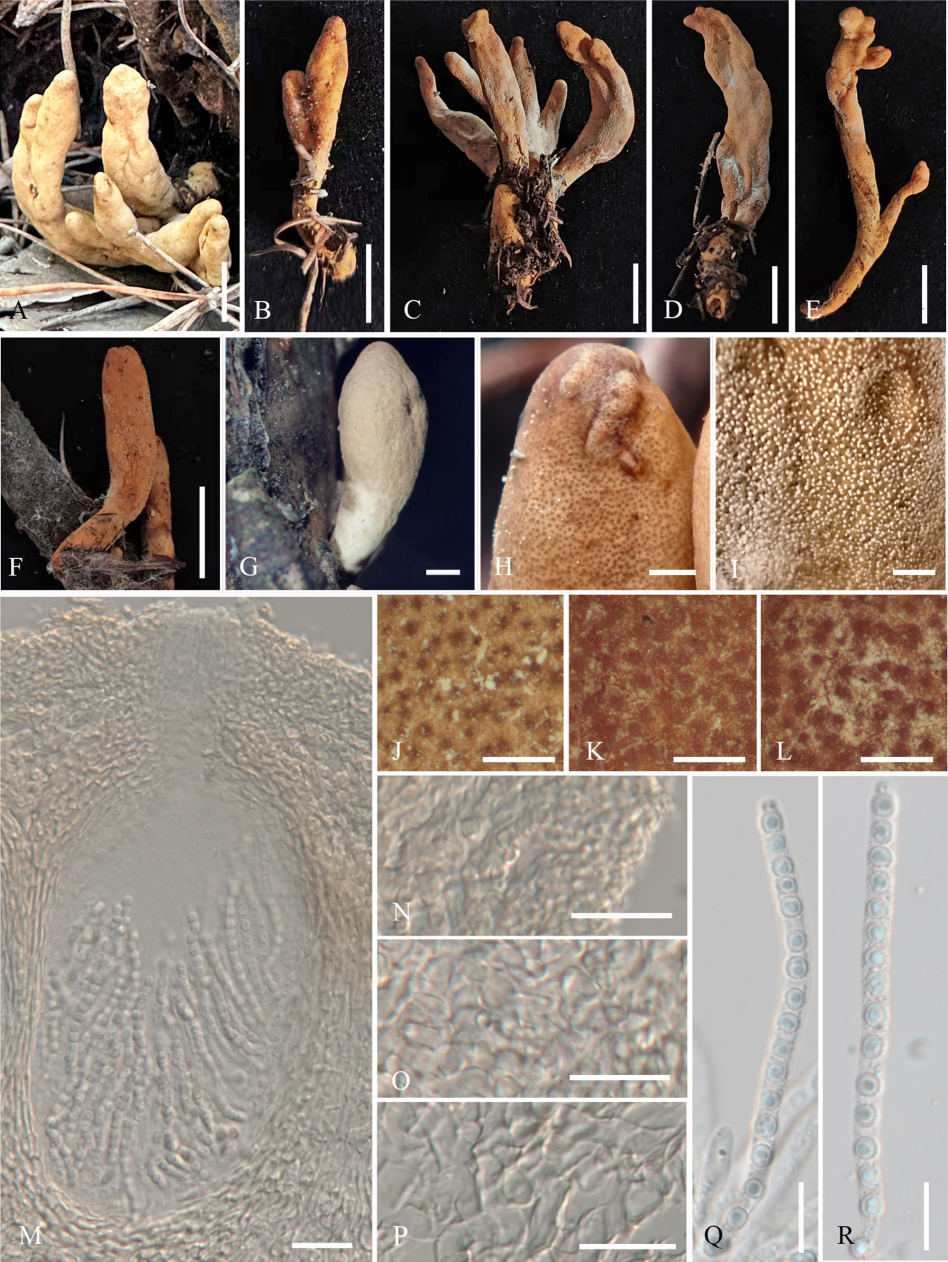
Sexual morph of *Trichoderma fulviclavum* (HMJAU 34754). **A**. Fresh stromata; **B–I**. Dry stromata; **J**. Surface of mature stromata; **K**. Stromatal surface after reconstitution in water; **L**. Stromatal surface in 3% KOH after reconstitution in water; **M**. Perithecium in section; **N**. Cortical and subcortical tissue in section; **O**. Subperithecial tissue in section; **P**. Basal tissue of stroma in section; **Q, R**. Asci with ascospores. Scale bars: 1 cm (**A–F**); 1 mm (**G, H**); 0.5 mm (**I**); 0.25 mm (**J–L**); 20 µm (**M–P**); 10 µm (**Q, R**).

In section stroma, ***perithecia*** flask-shaped, elliptical or globose (83–)93–193(–207) × 75–170(–193) µm (*n* = 30). ***Ostioles*** (30–)39–57(–62) µm long (*n* = 20), 17–35(–40) µm wide at the apex (*n* = 20). ***Peridium*** brown, (6.3–)7.4–8.2(–9.1) µm wide at the base and sides (*n* = 20). Cortical tissue of ***textura angularis***, cells hyaline, thick-walled, (2.8–)3.9–5.9 × (1.6–)2–4.1(–4.6) μm (*n* = 20); subcortical tissue of ***textura angularis*** mixed with ***textura intricata***, cells hyaline, thin-walled, 4.4–8(–10.2) × 3.1–4.3(–4.8) μm (*n* = 20), hyphae hyaline, thin-walled, 1.8–4.4 μm wide (*n* = 20); subperithecial tissue of ***textura epidermoidea***, cells hyaline, thin-walled, 3.5–8(–11) μm wide (*n* = 20); tissue at the base of ***textura intricata***, cells hyaline, thin-walled, 3.9–8.1 μm wide (*n* = 20). ***Asci*** 60–75(–80) × (2.8–)3.2–4.4 µm (*n* = 20). ***Ascospores*** monomorphic, hyaline, verruculose, cells subglobose to globose, 2.6–3.9 × 2.4–3.5 µm (*n* = 30), l/w 0.9–1.1(–1.3).

**Figure 6.**
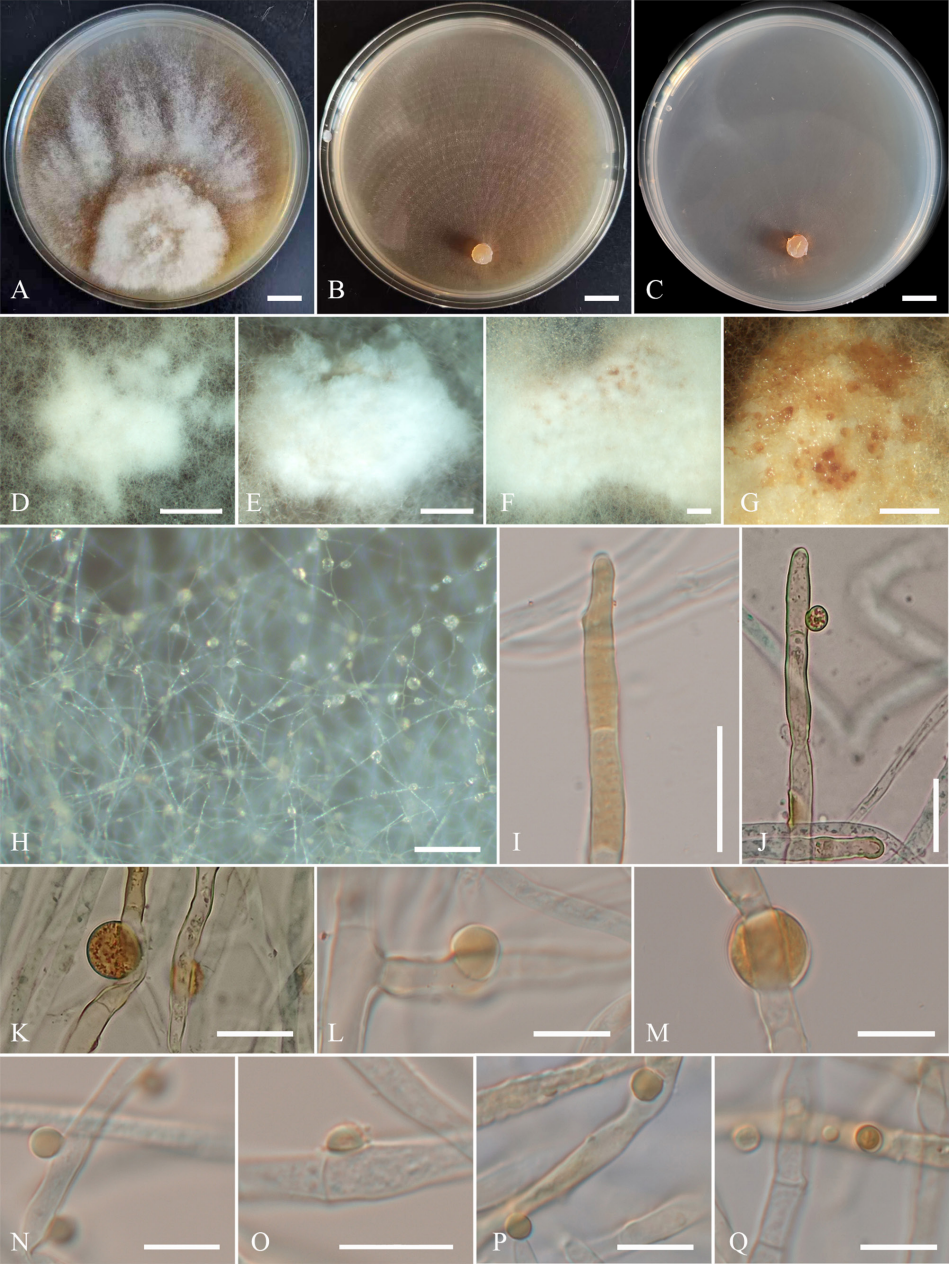
Colonies and microscopic characteristics of *Trichoderma fulviclavum* (Holotype CGMCC 7.673). **A–C**. Colonies at 25 °C on media (**A**PDA 25 d, **B**CMD 30 d, **C**SNA 42 d); **D–G**. Pustule on PDA; **H**. Exudates on aerial hyphae; **I**. Hyphal tip discoloration; **J–Q**. Chlamydoconidia sessile on hyphae. Scale bars: 1 cm (**A–C**); 250 μm (**D, H**); 1 mm (**E–G**); 10 µm (**I–Q**).

##### Cultures and asexual morph.

On PDA at 25 °C, colony radius 1–3 mm after 72 h, the plate fully covered after 3 weeks. Colonies initially white, gradually turning brown with age, with aerial hyphae densely developed at the colony base; exudate droplets present on aerial hyphae, small, light brown to brown, and evenly distributed. After 30 days, ***chlamydoconidia*** observed, brown, globose to subglobose, sessile on hyphae, (3–)3.3–10.7(–11.9) × (3.5–)3.9–11.3(–12.3) µm (*n* = 30), l/w 1–1.3(–1.5). Conidiophores and conidia not observed. Odor absent; brown pigment present.

On CMD at 25 °C, colony radius 1–2 mm after 72 h, with the plate fully covered after 4 weeks. Colonies white, forming a dense concentric annular pattern, with rare aerial hyphae. Conidia and chlamydospores not observed after 4 weeks. Odor absent; brown pigment present.

On SNA at 25 °C, colony radius 1–2 mm after 72 h, reaching 34–37 mm after 40 days. Colonies white, faint, forming a dense concentric annular pattern, without aerial hyphae. Conidiation not observed after 40 days. Odor absent; brown pigment present.

##### Additional specimens examined.

China • Jilin Province, Changchun City, Shuangyang District, 43°21'3"N, 125°53'41"E, 365 m alt., on pine branches, 31 Jul. 2025, Z.X. Zhu, G.S. Jin, J. Ji, G.L. Zhao, X.Z. Li 2196, HMJAU 34758, ex-type culture HMJAU 34759.

##### Notes.

*Trichoderma fulviclavum* is characterized by clavate stromata, extremely slow vegetative growth, absence of conidiation, and the production of brown chlamydoconidia. Morphologically, it resembles clavate-stromatic species such as *T. alutaceum*, *T. leucopus*, *T. seppoi*, and *T. clavaticapitatum*, all of which share the absence of a color change in 3% KOH (Jaklitsch 2011). In its asexual morph, *T. fulviclavum* exhibits an exceptionally slow growth rate on PDA (1–3 mm after 72 h), comparable to members of the Psychrophilum clade, including *T. psychrophilum* (< 1 mm after 72 h), *T. rhododendri* (4–5 mm after 72 h), *T. balearicum* (19–22 mm after 72 h), *T. crystalligenum* (8–10 mm after 72 h), *T. calamagrostidis* (9–16 mm after 72 h), and *T. oligosporum* (9–12 mm after 72 h) (Jaklitsch 2011; Zhu and Zhuang 2015; Zeng et al. 2024). Conidia were not observed in *T. fulviclavum*, similar to *T. rhododendri*; instead, chlamydoconidia were observed to be sessile on hyphae, a rarely reported character in *Trichoderma*.

Phylogenetically, *T. fulviclavum* clusters closely with *T. rhododendri* (CBS 119288) and *T. psychrophilum*, whereas it is distantly related to *T. leucopus*, *T. alutaceum*, and other clavate-stromatic species; detailed morphological distinctions are provided in Table 3. Notably, clavate stromata have not previously been reported in the Psychrophilum clade.

**Table 3.** Morphological comparisons of *Trichoderma fulviclavum* with *T. rhododendri* and *T. psychrophilum*. Traits with significant differences are highlighted in a dark color. A dash (–) indicates that the character is not reported.

Character		*T. rhododendri* (Jaklitsch 2011)	*T. psychrophilum* (Jaklitsch 2011)	*T. fulviclavum* (this study)
Stromata	Color	Yellowish to pale orange	Brownish yellow or grayish yellow	Pale white to brown
	Color in 3% KOH	Not changing color	Not changing color	Not changing color
	Shape	Pulvinate or discoid	Pulvinate or semi to subglobose	Clavate
	Diameter (mm)	(0.7–)1.3–2.6(–3.0) × (0.7–)1.0–1.7	(1.3–)1.5–3.3(–5.1) × (0.9–)1.2–2.4(–3.2)	up to 57
	Thickness (mm)	(0.2–)0.3–0.6	(0.5–)0.6–1.4(–2.0)	12–15
Ostioles	Color	Yellow–ochre	Orange to nearly red	Brown
	Length (μm)	(69–)86–111(–126)	(67–)75–98(–116)	(30–)39–57(–62)
	Width at the apex (μm)	(32–)36–54(–75)	(28–)30–45(–60)	17–35(–40)
Perithecia	Color of peridium	Hyaline to pale yellowish	Pale yellowish	Hyaline
	Length (μm)	(170–)200–245(–270)	(160–)200–250(–290)	(83–)93–193(–207)
	Width (μm)	(115–)130–200(–235)	(90–)130–200(–215)	75–170(–193)
	Asci length (μm)	(97–)100–116(–135)	(85–)100–130(–150)	60–75(–80)
	Asci width (μm)	(4.5–)5.0–6.0(–6.5)	(5.0–)5.5–6.2(–7.0)	(2.8–)3.2–4.4
Ascospore	Shape and ornamentation	Verruculose, cells subglobose, ellipsoidal or wedge-shaped	Verruculose, cells subglobose, ellipsoidal or wedge-shaped	Verruculose, cells subglobose to globose
	Color	Hyaline	Hyaline	Hyaline
	Distal length (μm)	(3.8–)4.0–5.0(–5.5)	(4.0–)4.5–5.7(–6.7)	2.6–3.9
	Distal width (μm)	(3.3–)3.5–4.0(–4.3)	(3.7–)4.0–4.5(–5.0)	2.4–3.5
	Distal l/w ratio	(1.0–)1.1–1.3(–1.4)	(1.0–)1.1–1.4(–1.7)	0.9–1.1(–1.3)
	Proximal length (μm)	(4.0–)4.5–5.5(–6.0)	(4.5–)5.2–6.5(–7.8)	–
	Proximal width (μm)	(2.7–)3.0–3.5(–4.0)	(3.0–)3.3–4.0(–4.5)	–
	Proximal l/w ratio	(1.1–)1.4–1.7(–1.8)	(1.2–)1.4–1.8(–2.2)	–
Colony radius after 72 h at 25 °C (mm)	CMD	0.2–1.5	0.2–1.5	1–2
	PDA	<1	<1	1–3
	SNA	1–2	1–2	1–2
Phialides	Length (μm)	–	(6–)7–12(–19)	–
	Width (μm)	–	(2.3–)2.8–3.5(–4.5)	–
	l/w ratio	–	(1.8–)2.3–4.0(–5.8)	–
	Basal width (μm)	–	(1.5–)2.0–2.8(–3.5)	–
Conidia	Shape and ornamentation		Ellipsoidal to oblong, smooth, eguttulate, or finely multiguttulate	
	Color	–	Hyaline	–
	Length (μm)	–	(3.2–)3.8–5.3(–7.0)	–
	Width (μm)	–	(2.3–)2.5–3.0(–3.7)	–
	l/w ratio	–	(1.3–)1.4–2.0(–2.5)	–
Chlamydospores/Chlamydoconidia	Length (μm)	(6–)7–12(–15)	–	(3–)3.3–10.7(–11.9)
	Width (μm)	(5–)6–9(–11)	–	(3.5–)3.9–11.3(–12.3)
	l/w ratio	(0.7–)1.0–1.7(–2.1)	–	1–1.3(–1.5)
Odor and pigment		Odor Absent, yellow-brown pigment	Absent	Odor Absent, yellow brown pigment

#### Trichoderma liaoningense

Taxon classificationFungiHypocrealesHypocreaceae

G.S. Jin & Z.X. Zhu sp. nov.

3CCAD45E-8242-5903-9732-A0B235ABD4CE

859961

Fig. 7

##### Etymology.

Named after Liaoning Province, China, where the species was collected.

##### Diagnosis.

Rapidly growing on PDA, forming dense colonies with abundant pale green to dark green pustules; phialides ampulliform; conidia obovate to subglobose.

##### Typification.

China • Liaoning Province, Shenyang city, Shenbei road, 41°55'30"N, 123°37'20"E, 1261 m alt., on twig, 20 Sep. 2024, Z.X. Zhu, G.S. Jin, J. Ji, G.L. Zhao, X.Z. Li 1914 (Holotype HMJAU 34752, ex-type culture CGMCC 7.671).

##### Sexual morph.

— Unknown.

##### Cultures and asexual morph.

On PDA at 25 °C, colony radius 57–60 mm after 72 h, with the plate fully covered after 4–5 days. Colonies white, with conspicuous aerial hyphae. Conidiation appearing after 72 h; pustules abundant, white when young, becoming dark green with age, fully covering the plate after 10 days. ***Conidiophores*** trichoderma-like, divergent in whorls of 2–4 phialides or with solitary phialides along the main axis. ***Phialides*** lageniform, (8.5–)9.5–13.2(–18.3) × (2.5–)2.8–3.7(–4.1) μm (*n* = 20), l/w (2.6–)2.9–4.3(–5.6), 1.7–2.6(–2.8) μm wide at the base (*n* = 20). ***Conidia*** hyaline when young, becoming green during growth, obovate, (3.4–)3.6–4.4(–4.9) × (2.5–)2.6–3(–3.2) μm (*n* = 20), l/w 1.2–1.5(–1.8). Odor absent; no pigment observed.

**Figure 7.**
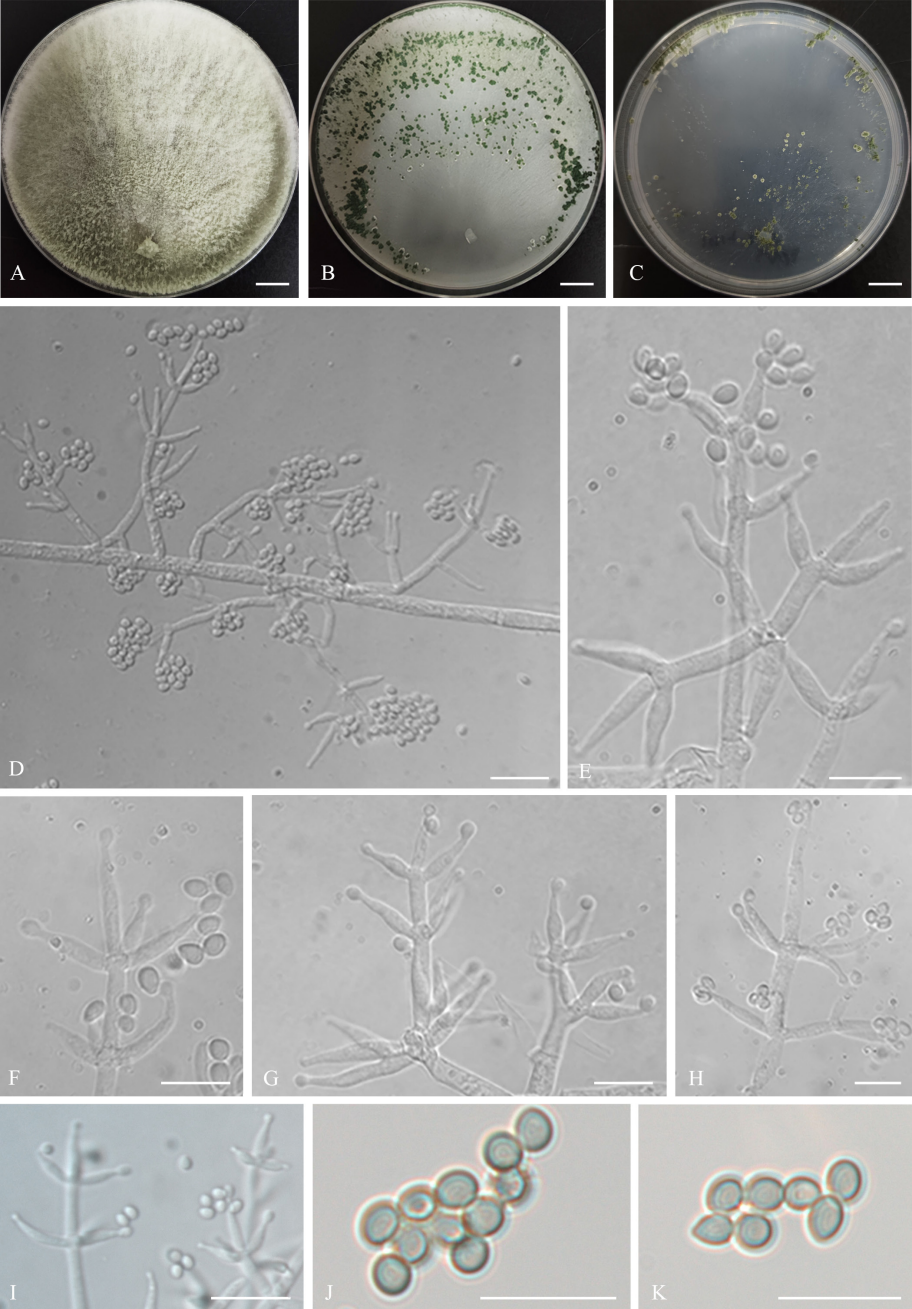
Colonies and microscopic characteristics of *Trichoderma liaoningense* (Holotype CGMCC 7.671). **A–C**. Colonies after 10 d at 25 °C on media (**A**PDA, **B**CMD, **C**SNA); **D–I**. Conidiophores and phialides; **J, K**. Conidia. Scale bars: 1 cm (**A–C**); 20 μm (**D**); 10 μm (**E–K**).

On CMD at 25 °C, colony radius 68–70 mm after 72 h, with the plate fully covered after 4 days. Colonies white, with inconspicuous aerial hyphae. Conidiation appearing after 4 days; pustules abundant, white to dark green, irregularly disposed in a broad concentric ring. Odor absent; no pigment observed.

On SNA at 25 °C, colony radius 40–43 mm after 72 h, with the plate fully covered after 7 days. Colonies white, with rare aerial hyphae. Conidiation appearing after 6–7 days; pustules zonate, spreading at the colony margin and near the center. Odor absent; no pigment observed.

##### Notes.

*Trichoderma liaoningense* is morphologically similar to *T. alni*, as both species are characterized by dense, thick mycelium on PDA with pale green conidiation and by green conidiation developing at the distal margin of the colony on CMD (Jaklitsch 2009). As in other members of the Brevicompactum clade, such as *T. brevicompactum* and *T. protrudens*, *T. liaoningense* forms pustules around the colony margin and in discrete patches on SNA and CMD. However, *T. liaoningense* exhibits a faster growth rate (57–60 mm on PDA and 68–70 mm on SNA) than *T. brevicompactum* (30–50 mm on PDA, ca. 35 mm on SNA) and *T. protrudens* (30–38 mm on PDA and 30–41 mm on SNA). In addition, *T. liaoningense* shares obovate conidia with *T. protrudens* (Degenkolb et al. 2008). Detailed morphological comparisons are provided in Table 4.

**Table 4.** Morphological comparisons of *Trichoderma liaoningense* with *T. brevicompactum* and *T. protrudens*. Traits with significant differences are highlighted in a dark color. A dash (–) indicates that the character is not reported.

Character		*T. brevicompactum* (Degenkolb et al. 2008)	*T. protrudens* (Degenkolb et al. 2008)	*T. liaoningense* (this study)
Colony radius after 72 h at 25 °C (mm)	CMD	–	–	68–70
	PDA	30–50	30–38	57–60
	SNA	35	30–41	40–43
Phialides	Length (μm)	(3.7–)4.7–6.7(–15.0)	(4.0–)5.5–9.0(–12.0)	(8.5–)9.5–13.2(–18.3)
	Width (μm)	(2.5–)3.0–3.7(–4.5)	(2.2–)2.5–3.0(–3.2)	(2.5–)2.8–3.7(–4.1)
	l/w ratio	(1.1–)1.3–2.2(–5.1)	(1.4–)1.8–3.4(–5.0)	(2.6–)2.9–4.3(–5.6)
	Basal width (μm)	(1.3–)2.0–2.7(–3.7)	(1.5–)1.7–2.7(–3.2)	1.7–2.6(–2.8)
Conidia	Shape	Subglobose	Subglobose to ovoidal	Obovate
	Color	Yellowish green	Green	Green
	Length (μm)	(2.2–)2.7–3.0(–3.7)	(2.5–)2.7–3.2(–3.5)	(3.4–)3.6–4.4(–4.9)
	Width (μm)	(2.0–)2.2–2.7(–3.0)	(2.2–)2.5–2.7(–3.0)	(2.5–)2.6–3(–3.2)
	l/w ratio	(0.9–)1.1–1.3(–1.4)	1.0–1.3(–1.5)	1.2–1.5(–1.8)
Chlamydospores	Length (μm)	(5.7–)8.0–11.0(–13.5)	–	–
	Width (μm)	(5.0–)6.5–9.2(–10.2)	–	–
Odor and pigment		Absent	Absent	Absent

Phylogenetically, *T. liaoningense* forms a distinct lineage within the Brevicompactum clade and differs from *T. protrudens* (DIS 119F) by 34 nucleotide substitutions in the 848 bp *rpb*2 locus, corresponding to an overall sequence similarity of approximately 96%.

#### Trichoderma parvostromatum

Taxon classificationFungiHypocrealesHypocreaceae

G.S. Jin & Z.X. Zhu sp. nov.

7375BC59-CD82-54BF-9222-86F8D64C63B4

859959

Figs 8, 9

##### Etymology.

The epithet *parvostromatum* is derived from the Latin *parvus* (small) and *stroma*, referring to the small-sized stromata of this species.

##### Diagnosis.

Stromata small, scattered, light brown, with small perithecia; ascospores hyaline and small. Colonies on PDA persistently white, with abundant aerial hyphae after prolonged incubation; conidia hyaline.

##### Typification.

China • Hubei Province, Shennongjia forestry district, Muyu Town, 31°29'5"N, 110°22'52"E, 129 m alt., on decaying wood, 12 Jul. 2024, Z.X. Zhu, G.S. Jin, J. Ji, G.L. Zhao, X.Z. Li, 1627 (Holotype HMJAU 34749, ex-type culture CGMCC 7.670).

##### Sexual morph.

***Stromata*** scattered, discrete, pulvinate, centrally attached, with margins more or less free, 0.2–1.2 mm diam, light brown, changing to orange in KOH. Stroma surface plane, smooth or slightly wrinkled, perithecial protuberances not evident, ostiolar brownish yellow or brown.

**Figure 8.**
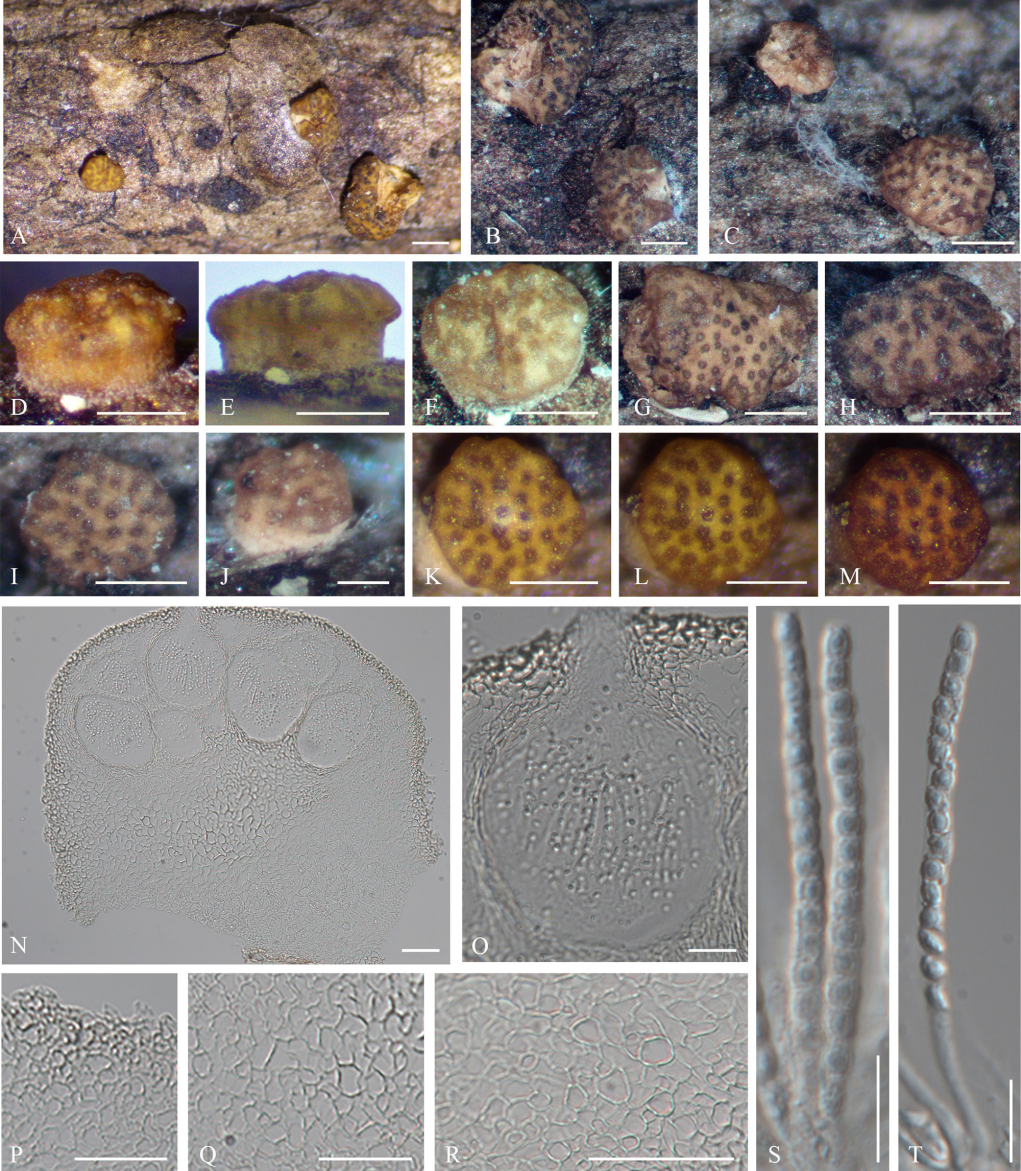
Sexual morph of *Trichoderma parvostromatum* (HMJAU 34749). **A–K**. Dry mature stromata; **L**. Mature stromata after reconstitution in water; **M**. Mature stromata in 3% KOH after reconstitution in water; **N**. Stromata in section; **O**. Perithecium in section; **P**. Cortical and subcortical tissue in section; **Q**. Subperithecial tissue in section; **R**. Basal tissue of stroma in section; **S, T**. Asci with ascospores. Scale bars: 0.5 mm (**A**); 0.25 mm (**B–I, K–M**); 0.1 mm (**J**); 50 µm (**N, P–R**); 20 µm (**O**); 10 µm (**S, T**).

In section stroma, ***perithecia*** flask-shaped or globose (100–)120–130(–150) × (84–)93–100(–110) µm (*n* = 20). ***Ostioles*** 31–39 µm long (*n* = 20), plane or projecting to 8 µm, 23–25 µm wide at the apex. ***Peridium*** hyaline, (4.5–)5.5–8.2(–10) µm wide at the base and sides (*n* = 20). Cortical tissue of ***textura angularis***, cells hyaline to light brown, thick-walled, (3.5–)5.4–9.6(–11.4) × (1.9–)2.6–4.9(–5.5) μm (*n* = 20); subcortical tissue of ***textura angularis*** mixed with ***textura intricata***, cells hyaline, thin-walled, (4.3–)4.9–6.8(–8.7) × (4.5–)5.2–12.3(–14.9) μm (*n* = 20), hyphae hyaline, thin-walled, 2–4.3 μm wide (*n* = 20); subperithecial tissue of ***textura angularis***, cells hyaline, thin-walled, 12–20(– 21.8) × (7.1–)8.4–12.4(–13.7) μm (*n* = 20); tissue at the base of ***textura angularis***, cells hyaline, thin-walled, (4.4–)5.5–9.1(–11.6) × (3–)5–7.9 μm (*n* = 20). ***Asci*** (42–)45–53(–56) × (2.8–)3–4(–4.3) µm (*n* = 20). ***Ascospores*** dimorphic, hyaline, smooth, distal cell subglobose to globose, (2–)2.3–3(–3.5) × (1.6–)1.8–2.2(–3) µm, l/w (0.7–)0.9–1.1(–1.3) (*n* = 20), proximal cell oblong or subglobose, 2.9–3.6(–4.2) × 2.1–2.7 µm (*n* = 20), l/w (1.1–)1.3–1.6(–1.9).

**Figure 9.**
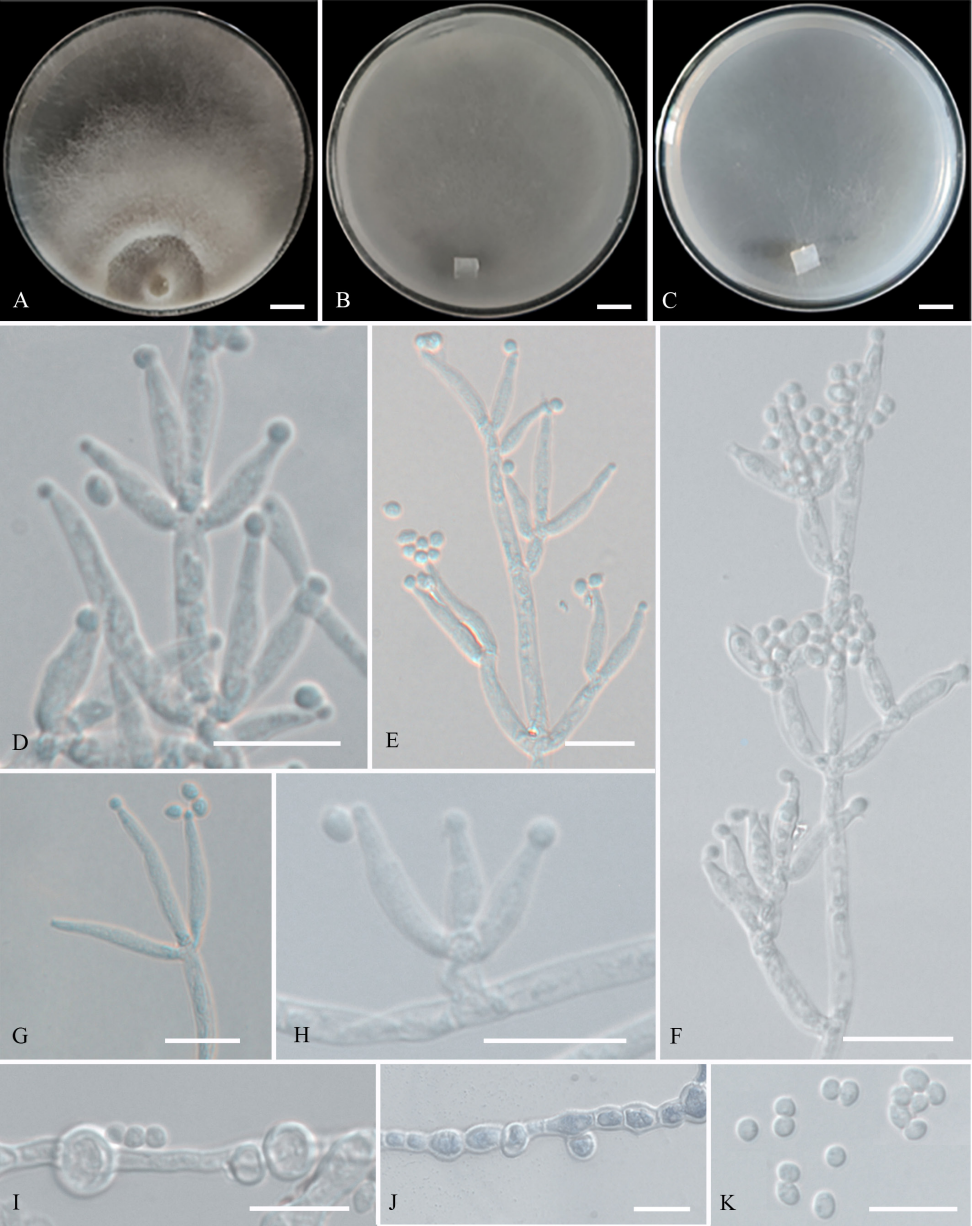
Colonies and microscopic characteristics of *Trichoderma parvostromatum* (Holotype CGMCC 7.670). **A–C**. Colonies after 3 weeks at 25 °C on media (**A**PDA, **B**CMD, **C**SNA); **D–H**. Conidiophores and phialides; **I, J**. Chlamydospore; **K**. Conidia. Scale bars: 1 cm (**A–C**); 10 μm (**D–K**).

##### Cultures and asexual morph.

On SNA at 25 °C, colony radius 23–28 mm after 72 h, with the plate fully covered after 15 days. Colonies white, with rare aerial hyphae. Conidiation appearing after 15 days. ***Conidiophores*** loosely disposed, verticillium-like. ***Phialides*** ampulliform, divergent in whorls of 2–4 phialides or with solitary phialides along the main axis, (6.3–)7.7–12.7(–18.8) × (1.5–)1.8–2.5(–2.9) μm (*n* = 20), l/w (2–)2.6–4.3(–5.6), (1.3–)1.8–2.3(–2.8) μm wide at the base (*n* = 20). ***Conidia*** hyaline, subglobose to globose, 2.1–3.4(–3.8) × 1.7–2.2(–2.4) μm (*n* = 20), l/w 1.1–1.6(–1.8). ***Chlamydospores*** numerous, terminal, and intercalary on hyphae, globose or ellipsoid; intercalary chlamydospores oblong or ellipsoidal, (4.4–)5–7.3(–8.2) × (3.7–)5–6.5(–7) μm (*n* = 20), l/w 1–1.5(–1.7). Odor absent; no pigment observed.

On PDA at 25 °C, colony radius 24–27 mm after 72 h, with the plate fully covered after 20 days. Colonies white, with velvety aerial hyphae forming isolated tufts or surrounding the base. Conidiation rarely appearing after 20 days; pustules rare, white. Chlamydospores numerous, globose or ellipsoid. Odor absent; no pigment observed.

On CMD at 25 °C, colony radius 41–45 mm after 72 h, with the plate fully covered after 10 days. Colonies white, with rare aerial hyphae. Conidiation appearing after 15 days; pustules absent. Chlamydospores numerous, globose or ellipsoid. Odor absent; no pigment observed.

##### Notes.

Morphologically, *Trichoderma parvostromatum* is similar to *T. semiorbis* and *T. hunua* in producing brown stromata. However, *T. parvostromatum* is distinguished by its markedly smaller stromata (0.2–1.2 mm) compared with those of *T. semiorbis* (1.5–4 mm) and *T. hunua* (2.5–4 mm). The perithecia of *T. parvostromatum* are also smaller [(100–)120–130(–150) μm] than those of *T. semiorbis* [(224–)268–287(–318) μm] and *T. hunua* (300–500 μm). In addition, *T. parvostromatum* possesses smooth and smaller ascospores [(2–)2.3–3(–3.5) × (1.6–)1.8–2.2(–3) μm], whereas the ascospores of *T. semiorbis* and *T. hunua* are echinulate and distinctly larger [(4.7–)5.5–6.5(–8.5) × (3.5–)3.7–4.5(–5.2) μm and 6–8 × 4–5 μm, respectively]. In the asexual morph, *T. parvostromatum* produces hyaline conidia, in contrast to the green conidia observed in *T. semiorbis* and *T. hunua* (Jaklitsch 2011). Detailed morphological distinctions among these species are summarized in Table 5.

**Table 5.** Morphological comparisons of *Trichoderma parvostromatum* with *T. hunua* and *T. semiorbis*. Traits with significant differences are highlighted in a dark color. A dash (–) indicates that the character is not reported.

Character		*T. hunua* (Dingley 1952)	*T. semiorbis* (Chaverri et al. 2003)	*T. parvostromatum* (this study)
Stromata	Color	Vinaceous brown	Light brown	Brownish yellow or brown
	Color in 3% KOH	–	Not changing color	Red brown
	Shape	Irregular, pulvinate	Scattered, discrete, pulvinate	Pulvinate
	Diameter (mm)	2.5–4	1.5–4	0.2–1.2
	Thickness (mm)	1–1.25	–	0.2–0.3
Ostioles	Color	Vinaceous brown	Viscid dots	Brownish yellow or brown
	Length (μm)	75–100	(59–)84–93(–109)	31–39
	Width at the apex (μm)	–	–	23–25
Perithecia	shape	Oval or pyriform	Subglobose	Oval or pyriform
	Color of peridium	Hyaline	–	Hyaline
	Length (μm)	300–500	(224–)268–287(–318)	(100–)120–130(–150)
	Width (μm)	250	(135–)160–177(–202)	(84–)93–100(–110)
	Asci length (μm)	100–180	(80–)102–107(–142)	(42–)45–53(–56)
	Asci width (μm)	4–5	(5.7–)7.5–7.7(–10.0)	(2.8–)3–4(–4.3)
Ascospore	Shape and ornamentation	Echinulate, cells globose, oval, or pyriform	Finely but densely spinulose, cells conical to wedge-shaped,	Smooth, cells subglobose to globose
	Color	Lightly pigmented yellow	Hyaline	Hyaline
	Distal length (μm)	6–8	(4.7–)5.5–6.5(–8.5)	(2–)2.3–3(–3.5)
	Distal width (μm)	4–5	(3.5–)3.7–4.5(–5.2)	(1.6–)1.8–2.2(–3)
	Distal l/w ratio	–	–	(0.7–)0.9–1.1(–1.3)
	Proximal length (μm)	5–6	(4.7–)5.2–6.7(–9.0)	2.9–3.6(–4.2)
	Proximal width (μm)	–	(2.7–)3.5–4.2(–5.2)	2.1–2.7
	Proximal l/w ratio	–	–	(1.1–)1.3–1.6(–1.9)
Colony radius after 72 h at 25 °C (mm)	CMD	–	–	41–45
	PDA	–	18–30	24–27
	SNA	–	10–17	23–28
Phialides	Length (μm)	5–10	(3.2–)4.5–6.5(–8.2)	(6.3–)7.7–12.7(–18.8)
	Width (μm)	2.5–5.5	(2.7–)3.0–3.7(–4.5)	(1.5–)1.8–2.5(–2.9)
	l/w ratio	–	(1.0–)1.3–2.0(–2.7)	(2–)2.6–4.3(–5.6)
	Basal width (μm)	–	(1.5–)2.0–3.0(–4.2)	(1.3–)1.8–2.3(–2.8)
Conidia	Shape	Globose	Oblong	Subglobose to globose
	Color	Olivaceous green	Green	Hyaline
	Length (μm)	3–4	3.5–4.5(–7.0)	2.1–3.4(–3.8)
	Width (μm)	–	(1.7–)2.2–2.7(–3.0)	1.7–2.2 (–2.4)
	l/w ratio	–	(1.3–)1.6–1.7(–2.5)	1.1–1.6(–1.8)
Chlamydospores	Length (μm)	–	(1.2–)1.5–2.5(–2.7)	(4.4–)5–7.3(–8.2)
	Width (μm)	–	–	(3.7–)5–6.5(–7)
	l/w ratio	–	–	1–1.5
Odor and pigment		–	Absent	Absent

Phylogenetically, *T. parvostromatum* is resolved as a distinct lineage within the Semiorbis clade and is closely related to *T. hunua* and *T. semiorbis*. The *rpb*2 sequence similarity between *T. parvostromatum* and *T. hunua* (CBS 238.63) is 96%, and the similarity between *T. parvostromatum* and *T. semiorbis* is 95%, with 30 and 32 bp differences across 893 bp and 836 bp alignments, supporting its recognition as a distinct species.

## Discussion

In this study, 209 *Trichoderma* isolates from decaying wood across five provinces in China were examined, resulting in the recognition of 25 species, including five species newly described herein. Although several of the newly recognized taxa are currently represented by single isolates, their recognition is supported by distinct morphological characteristics and well-resolved phylogenetic placements.

Phylogenetic analyses based on *rpb*2 and *tef*1-α placed the five new species in five major clades, Brevicompactum, Harzianum, Longibrachiatum, Psychrophilum, and Semiorbis—consistent with previously established phylogenetic frameworks (Jaklitsch 2009; Jaklitsch 2011; Cai and Druzhinina 2021). All five species were isolated from decaying wood, indicating a saprotrophic life strategy associated with lignocellulosic substrates in forest ecosystems. These taxa were collected from four different provinces in China, reflecting a geographically dispersed distribution. The newly described *Trichoderma* species are distributed across several phylogenetically distinct clades, including economically important groups such as the Harzianum clade (Zimand et al. 1996), which contains widely used biocontrol agents, and less species-rich clades such as Semiorbis, Brevicompactum, and Psychrophilum (Chaverri et al. 2003; Degenkolb et al. 2008; Jaklitsch 2011), highlighting decaying wood as an important reservoir of *Trichoderma* diversity and suggesting that substantial taxonomic diversity remains to be discovered.

Among the five *Trichoderma* species identified in this study, *T. fulviclavum* is particularly noteworthy due to its production of clavate stromata, a feature traditionally associated with a limited number of taxa within the genus. Historically, most Hypocreaceae species bearing clavate stromata were classified in the genus *Podostroma* (Saccardo and Roumeguère 1885; Doi 1978; Jaklitsch et al. 2008); however, following the implementation of the “one fungus, one name” principle in the 2011 revision of the International Code of Nomenclature for Algae, Fungi, and Plants, the majority of these taxa were transferred to *Trichoderma* (Bissett et al. 2015). Within *Trichoderma*, species with clavate stromata are predominantly assigned to the Viride clade, including *T. nybergianum*, *T. seppoi*, *T. leucopus*, and *T. clavaticapitatum* (Jaklitsch 2011; Zeng et al. 2024), with *T. cornu-damae* placed in the Brevicompactum clade (Bissett et al. 2015).

In contrast, *T. fulviclavum* is placed in the Psychrophilum clade, in which clavate-stromatic taxa have not previously been reported. Species within this clade are characterized by relatively slow growth rates and reduced or absent conidiation in culture, traits that closely correspond to those observed in *T. fulviclavum*. Representative examples include *T. psychrophilum*, which grows less than 1 mm after 72 h on PDA (Jaklitsch 2011), *T. rhododendri* with a colony radius of 4–5 mm after 72 h (Jaklitsch 2011), as well as *T. crystalligenum* (Jaklitsch 2011), *T. oligosporum* (Zhu and Zhuang 2015), and *T. calamagrostidis* (Zeng et al. 2024), all of which exhibit slow vegetative growth and limited sporulation under standard culture conditions.

These observations indicate that stromatal morphology alone may be evolutionarily labile and insufficient for resolving phylogenetic relationships within *Trichoderma*. In contrast, physiological and developmental traits of the asexual morph—such as growth rate and conidiation capacity—appear to be more consistent at the clade level and may carry greater phylogenetic signal. The placement of *T. fulviclavum* therefore highlights the potential role of convergent evolution in shaping stromatal morphology and further expands the morphological diversity currently recognized within the Psychrophilum clade.

A widespread characteristic of *Trichoderma* species is the production of trichoderma-like conidia in their asexual morph (Chaverri et al. 2003). In *T. fulviclavum*, however, the asexual morph exhibits a relatively slow growth rate comparable to that of other members of the Psychrophilum clade. Species within this clade are typically characterized by pulvinate stromata and white-conidial anamorphs with more or less gliocladium-like conidiophores (Jaklitsch 2011). In contrast, no conidiophores or conidia were observed in *T. fulviclavum* on any of the culture media examined; instead, only chlamydoconidia were produced on PDA.

Chlamydoconidia have been reported in several fungal genera such as *Candida* and *Rhizopus* (Kianipour et al. 2018; Rocha et al. 2019), but they are rarely documented in *Trichoderma*. The occurrence of chlamydoconidia as the sole asexual propagules in *T. fulviclavum* therefore represents an unusual morphological condition within the genus. Despite the absence of conidiation, *T. fulviclavum* can be confidently assigned to *Trichoderma* based on the presence of typical hypocrealean sexual characters, its phylogenetic placement within the genus, and concordant sequence data from *rpb*2 and *tef*1-α. These observations broaden the known range of asexual morphologies within *Trichoderma* and further expand the morphological diversity currently recognized in the Psychrophilum clade. They also illustrate that conidiation, although common, is not an obligatory feature of the asexual stage in all *Trichoderma* species, emphasizing the importance of integrating morphological and molecular evidence when dealing with atypical taxa.

In addition to commonly used loci such as ITS, *rpb*2, and *tef*1-α (Cai and Druzhinina 2021), several additional protein-coding genes, including actin (*ACT*), calmodulin (*CAL*), β-tubulin (*tub*2), and GH 18 chitinase (*chi18-5*) (Druzhinina et al. 2010; Chaverri et al. 2015), have been employed in phylogenetic studies of *Trichoderma* and related fungi. However, these loci have been used less frequently in recent multilocus frameworks due to limited sequence availability and comparatively low resolution at the species level.

Although *rpb*2 and *tef*1-α remain among the most informative markers for species delimitation in *Trichoderma*, they also exhibit inherent limitations, particularly in species complexes characterized by recent divergence or weak phylogenetic signal (Cai and Druzhinina 2021). These challenges include incomplete representation of *rpb*2 sequences in public databases, high variability and inconsistent amplification of *tef*1-α, and difficulties in aligning homologous regions across distantly related taxa. In addition, outdated or poorly annotated sequences deposited in public repositories such as NCBI further complicate accurate species delimitation.

Given these limitations, genome-scale data provide access to numerous orthologous loci and can improve phylogenetic resolution and species delimitation. Since the first genome of *Trichoderma reesei* was published in 2008, genomic resources of the genus have expanded rapidly (Martinez et al. 2008; Schalamun and Schmoll 2022). As of December 2025, 224 genome assemblies representing more than 40 *Trichoderma* species are available in NCBI, providing a valuable resource for future phylogenomic studies.

With the increasing availability of genomic resources, future studies of *Trichoderma* will benefit from integrating genome-derived markers or whole-genome data to further improve species delimitation.

## Supplementary Material

XML Treatment for Trichoderma flavobritdaniae

XML Treatment for Trichoderma foliatum

XML Treatment for Trichoderma fulviclavum

XML Treatment for Trichoderma liaoningense

XML Treatment for Trichoderma parvostromatum

## Additional information

### Artificial Intelligence (AI) use

The authors accept full responsibility for the content of the manuscript, including the disclosure of any use of AI.

No AI tools were used in the preparation of this manuscript.

### Author ORCIDs

G.-S. Jin https://orcid.org/0009-0007-8683-7395

J. Ji https://orcid.org/0009-0006-5854-0407

G.-L. Zhao https://orcid.org/0009-0004-6586-5168

X.-Z. Li https://orcid.org/0009-0008-0360-2513

Y. Yu https://orcid.org/0009-0000-9546-4884

W.-Y. Zhuang https://orcid.org/0000-0002-1162-9678

Y. Li https://orcid.org/0000-0003-4719-7210

R.-Q. Ji https://orcid.org/0000-0002-4799-0270

Z.-X. Zhu https://orcid.org/0000-0002-8330-918X

### Data availability

All of the data that support the findings of this study are available in the main text or Supplementary Information.

## Supplementary materials

Supplementary material 1 Phylogenetic trees, based on the Maximum Likelihood analysis of the rpb2 dataset Data type zip Explanation note Phylogenetic tree, based on the Maximum Likelihood analysis of the rpb2 dataset. MLBP above 75% (left) and BIPP above 0.90 (right) are indicated at the nodes. New species proposed here are indicated in red, T = ex-type strain. https://binary.pensoft.net/file/1659683This dataset is made available under the Open Database License (http://opendatacommons.org/licenses/odbl/1.0/). The Open Database License (ODbL) is a license agreement intended to allow users to freely share, modify, and use this Dataset while maintaining this same freedom for others, provided that the original source and author(s) are credited. Guang-Shuo Jin, Zhao-Xiang Zhu

Supplementary material 2 Phylogenetic tree, based on the Maximum Likelihood analysis of the tef1-α dataset Data type zip Explanation note Phylogenetic tree, based on the Maximum Likelihood analysis of the tef1-α dataset. MLBP above 75% (left) and BIPP above 0.90 (right) are indicated at the nodes. New species proposed here are indicated in red, T = ex-type strain. https://binary.pensoft.net/file/1659684This dataset is made available under the Open Database License (http://opendatacommons.org/licenses/odbl/1.0/). The Open Database License (ODbL) is a license agreement intended to allow users to freely share, modify, and use this Dataset while maintaining this same freedom for others, provided that the original source and author(s) are credited. Guang-Shuo Jin, Zhao-Xiang Zhu

## References

[B1] Alwadai AS, Al Wahibi MS, Alsayed MF, Alshaikh NA, Perveen K, Elsayim R (2024) Molecular characterization of plant growth-promoting *Trichoderma* from Saudi Arabia. Scientific Reports 14: 23236. 10.1038/s41598-024-73762-5 PMC11457496 39369094

[B2] An XY, Cheng GH, Gao HX, Li XF, Yang Y, Li D, Li Y (2022) Phylogenetic analysis of *Trichoderma* species associated with green mold disease on mushrooms and two new pathogens on *Ganoderma sichuanense*. Journal of Fungi 8: 704. 10.3390/jof8070704 PMC9318549 35887460

[B3] Bissett J, Gams W, Jaklitsch W, Samuels GJ (2015) Accepted *Trichoderma* names in the year 2015. IMA Fungus 6: 263–295. 10.5598/imafungus.2015.06.02.02 PMC4681254 26734542

[B4] Cai F, Druzhinina IS (2021) In honor of John Bissett: authoritative guidelines on molecular identification of *Trichoderma*. Fungal Diversity 107: 1–69. 10.1007/s13225-020-00464-4

[B5] Cao ZJ, Qin WT, Zhao J, Liu Y, Wang SX, Zheng SY (2022) Three new *Trichoderma* species in harzianum clade associated with the contaminated substrates of edible fungi. Journal of Fungi 8: 1154. 10.3390/jof8111154 PMC9696741 36354921

[B6] Cao ZJ, Zhao J, Liu Y, Wang SX, Zheng SY, Qin WT (2024) Diversity of *Trichoderma* species associated with green mold contaminating substrates of *Lentinula edodes* and their interaction. Frontiers in Microbiology 14: 1288585. 10.3389/fmicb.2023.1288585 PMC10800798 38260891

[B7] Chaverri P, Branco-Rocha F, Jaklitsch W, Gazis R, Degenkolb T, Samuels GJ (2015) Systematics of the *Trichoderma harzianum* species complex and the re-identification of commercial biocontrol strains. Mycologia 107: 558–590. 10.3852/14-147 PMC4885665 25661720

[B8] Chaverri P, Castlebury LA, Overton BE, Samuels GJ (2003) *Hypocrea/Trichoderma*: species with conidiophore elongations and green conidia. Mycologia 95: 1100–1140. 10.1080/15572536.2004.11833023 21149016

[B9] Chen K, Zhuang WY (2016) *Trichoderma shennongjianum* and *Trichoderma tibetense*, two new soil-inhabiting species in the Strictipile clade. Mycoscience 57: 311–319. 10.1016/j.myc.2016.04.005

[B10] Chen K, Zhuang WY (2017) Discovery from a large-scaled survey of *Trichoderma* in soil of China. Scientific Reports 7: 9090. 10.1038/s41598-017-07807-3 PMC5567330 28831112

[B11] De Lima EM, Pereira Filho AM, Da Costa DP, Da França RF, Da Silva ELL, Santos MC, De Barros JA, De Souza CAF, Lima JRDS, Duda GP, Hammecker C, De Medeiros ÉV (2025) Potential of biochar inoculated with *Trichoderma* to improve soil chemical and biological properties in a regenerating area. Journal of Arid Environments 230: 105430. 10.1016/j.jaridenv.2025.105430

[B12] Degenkolb T, Dieckmann R, Nielsen KF, Gräfenhan T, Theis C, Zafari D, Chaverri P, Ismaiel A, Brückner H, von Döhren H, Thrane U, Petrini O, Samuels GJ (2008) The *Trichoderma brevicompactum* clade: a separate lineage with new species, new peptaibiotics, and mycotoxins. Mycological Progress 7: 177–219. 10.1007/s11557-008-0563-3

[B13] Dingley JM (1952) The Hypocreales of New Zealand. III. The genus *Hypocrea*. Transactions and Proceedings of the Royal Society of New Zealand 79: 323–337.

[B14] Doi Y (1978) Revision of the hypocreales with cultural observations. XI. Additional notes on *Hypocrea* and its allies in Japan. 1. Australian Journal of Psychology 33: 149–156.

[B15] Druzhinina IS, Kubicek CP, Komon-Zelazowska M, Belayneh Mulaw T, Bissett J (2010) The *Trichoderma harzianum* demon: complex speciation history resulting in coexistence of hypothetical biological species, recent agamospecies and numerous relict lineages. BMC Evolutionary Biology 10: 94. 10.1186/1471-2148-10-94 PMC2858147 20359347

[B16] García-Latorre C, Ruiz-Moyano S, Rodríguez A, Velázquez R, Poblaciones MJ, Hernández A (2025) From field to fork: the benefits of *Trichoderma* spp. in food quality and safety. Current Opinion in Food Science 63: 101286. 10.1016/j.cofs.2025.101286

[B17] Guzmán-Guzmán P, Etesami H, Santoyo G (2025) *Trichoderma*: a multifunctional agent in plant health and microbiome interactions. BMC Microbiology 25: 434. 10.1186/s12866-025-04158-2 PMC12255041 40652165

[B18] Hall T (2012) BioEdit v7.0.9: Biological sequence alignment editor for Win95/98/2K/XP/7.

[B19] Huang QR, Senanayake IC, Liu JW, Chen WJ, Dong ZY, Luo M (2024) *Trichoderma azadirachtae* sp. nov. from rhizosphere soil of *Azadirachta indica* from Guangdong Province, China. Phytotaxa 670: 148–160. 10.11646/phytotaxa.670.3.1

[B20] Jaklitsch WM, Poldmaa K, Samuels GJ (2008) Reconsideration of *Protocrea* (Hypocreales, Hypocreaceae). Mycologia 100: 962–984. 10.3852/08-101 PMC3077755 19202850

[B21] Jaklitsch WM (2009) European species of *Hypocrea* Part I. The green-spored species. Studies in Mycology 63: 1–91. 10.3114/sim.2009.63.01 PMC2757427 19826500

[B22] Jaklitsch WM (2011) European species of *Hypocrea* part II: species with hyaline ascospores. Fungal Diversity 48: 1–250. 10.1007/s13225-011-0088-y PMC3189789 21994484

[B23] Jaklitsch WM, Komon M, Kubicek CP, Druzhinina IS (2005) *Hypocrea voglmayrii* sp. nov. from the Austrian Alps represents a new phylogenetic clade in *Hypocrea/Trichoderma*. Mycologia 97: 1365–1378. 10.1080/15572536.2006.11832743 16722227

[B24] Jaklitsch WM, Voglmayr H (2012) *Hypocrea britdaniae* and *H. foliicola*: two remarkable new European species. Mycologia 104: 1213–1221. 10.3852/11-429 PMC4139776 22505436

[B25] Jaklitsch WM, Voglmayr H (2015) Biodiversity of *Trichoderma* (Hypocreaceae) in Southern Europe and Macaronesia. Studies in Mycology 80: 1–87. 10.1016/j.simyco.2014.11.001 PMC4779795 26955191

[B26] Kianipour S, Ardestani ME, Dehghan P (2018) Identification of *Candida albicans* and *Candida dubliniensis* species isolated from bronchoalveolar lavage samples using genotypic and phenotypic methods. Advanced Biomedical Research 7: 66. 10.4103/abr.abr_138_17 PMC5952538 29862215

[B27] Lagashetti A, Singh S, Singh PN (2023) *Trichoderma indica*: a new species in the Longibrachiatum clade from Western Ghats, India. Turkish Journal of Botany 47: 595–605. 10.55730/1300-008x.2787

[B28] Li J, Wu Y, Chen K, Wang Y, Hu J, Wei Y, Yang H (2018) *Trichoderma cyanodichotomus* sp. nov., a new soil-inhabiting species with a potential for biological control. Canadian Journal of Microbiology 64: 1020–1029. 10.1139/cjm-2018-0224 30199653

[B29] Li X, Liao Q, Zeng S, Wang Y, Liu J (2025) The use of *Trichoderma* species for the biocontrol of postharvest fungal decay in fruits and vegetables: Challenges and opportunities. Postharvest Biology and Technology 219: 113236. 10.1016/j.postharvbio.2024.113236

[B30] Liu JK, Maharachchikumbura SSN, McKenzie EHC, Cai L, Bhat JD, Jones EBG, Stadler M (2021) Special issue: a tribute to Kevin D. Hyde on his 65^th^ birthday. Fungal Diversity 106: 1–6. 10.1007/s13225-021-00473-x

[B31] Liu XF, Tibpromma S, Hughes A, Kandawatte T, Wijayawardene N, Dai DQ, Du TY, Elgorban A, Stephenson S, Suwannarach N, Xu J, Lu L, Xu RF, Maharachchikumbura S, Zhao C, Bhat DJ, Ym S, Karunarathna S, Mortimer P (2023) Culturable mycota on bats in central and southern Yunnan Province, China. Mycosphere 14: 497–662. 10.5943/mycosphere/14/1/7

[B32] Liu YJ, Whelen S, Hall BD (1999) Phylogenetic relationships among ascomycetes: evidence from an RNA polymerse II subunit. Molecular Biology and Evolution 16: 1799–1808. 10.1093/oxfordjournals.molbev.a026092 10605121

[B33] Ma Y, Zuohereguli K, Zhang L, Kang Y, Shi L, Xu H, Ruan Y, Wen T, Mei X, Dong C, Xu Y, Shen Q (2025) Soil microbial mechanisms to improve pear seedling growth by applying *Bacillus* and *Trichoderma*-amended biofertilizers. Plant, Cell and Environment 48: 3968–3980. 10.1111/pce.15395 39871496

[B34] Ma YK, Yang SJ, Li YT, Gao HX, Li YU, Zhu ZX (2024) *Trichoderma wuyiense* (Hypocreales, Ascomycota), a new species from Wuyi Mountains, China. Phytotaxa 635: 217–226. 10.11646/phytotaxa.635.3.3

[B35] Martinez D, Berka RM, Henrissat B, Saloheimo M, Arvas M, Baker SE, Chapman J, Chertkov O, Coutinho PM, Cullen D, Danchin EGJ, Grigoriev IV, Harris P, Jackson M, Kubicek CP, Han CS, Ho I, Larrondo LF, De Leon AL, Magnuson JK, Merino S, Misra M, Nelson B, Putnam N, Robbertse B, Salamov AA, Schmoll M, Terry A, Thayer N, Westerholm-Parvinen A, Schoch CL, Yao J, Barabote R, Nelson MA, Detter C, Bruce D, Kuske CR, Xie G, Richardson P, Rokhsar DS, Lucas SM, Rubin EM, Dunn-Coleman N, Ward M, Brettin TS (2008) Genome sequencing and analysis of the biomass-degrading fungus *Trichoderma reesei* (syn. *Hypocrea jecorina*). Nature Biotechnology 26: 553–560. 10.1038/nbt1403 18454138

[B36] Mishra N, Prasad P, Mahfooz S, Shivhare R, Verma P, Singh SP, Mishra S, Kumar Mishra S, Bajpai R, Mishra A (2024) Comprehensive analysis of *Trichoderma reesei* mediated CO_2_ stress attenuating responses and *de novo* transcriptome sequencing of rice flag leaf. Physiologia Plantarum 176: 1. 10.1111/ppl.14160

[B37] Nascimento Brito V, Lana Alves J, Sírio Araújo K, De Souza Leite T, Borges De Queiroz C, Liparini Pereira O, De Queiroz MV (2023) Endophytic *Trichoderma* species from rubber trees native to the Brazilian Amazon, including four new species. Frontiers in Microbiology 14: 1095199. 10.3389/fmicb.2023.1095199 PMC10151590 37143529

[B38] Nguyen L-T, Schmidt HA, Von Haeseler A, Minh BQ (2015) IQ-TREE: A fast and effective stochastic algorithm for estimating maximum-likelihood phylogenies. Molecular Biology and Evolution 32: 268–274. 10.1093/molbev/msu300 PMC4271533 25371430

[B39] Nirenberg H (1976) Untersuchungen über die Morphologische und Biologische Differenzierung in der *Fusarium*-Sektion Liseola. Mitteilungen aus der Biologischen Bundesanstalt für Land- und Forstwirtschaft (Berlin-Dahlem) 169.

[B40] Nylander J (2004) MrModeltest V2. Program distributed by the author. Bioinformatics 24: 581–583.

[B41] Pandey AK, Yadav S, Samota MK, Sharma HK, Roy S (2024) *Trichoderma harzianum* TIND02 upregulates the expression of pathogenesis-related genes and enzymes and enhances gray blight resistance in tea. Pesticide Biochemistry and Physiology 205: 106115. 10.1016/j.pestbp.2024.106115 39477576

[B42] Perera RH, Hyde KD, Jones EBG, Maharachchikumbura SSN, Bundhun D, Camporesi E, Akulov A, Liu JK, Liu ZY (2023) Profile of Bionectriaceae, Calcarisporiaceae, Hypocreaceae, Nectriaceae, Tilachlidiaceae, Ijuhyaceae fam. nov., Stromatonectriaceae fam. nov. and Xanthonectriaceae fam. nov. Fungal Diversity 118: 95–271. 10.1007/s13225-022-00512-1

[B43] Pollard-Flamand J, Boulé J, Hart M, Úrbez-Torres JR (2022) Biocontrol activity of *Trichoderma* species isolated from grapevines in british columbia against botryosphaeria dieback fungal pathogens. Journal of Fungi 8: 409. 10.3390/jof8040409 PMC9030288 35448640

[B44] Qiao M, Du X, Zhang Z, Xu J, Yu Z (2018) Three new species of soil-inhabiting *Trichoderma* from southwest China. MycoKeys 44: 63–80. 10.3897/mycokeys.44.30295 PMC6303281 30595658

[B45] Qin WT, Zhuang WY (2016a) Four new species of *Trichoderma* with hyaline ascospores from central China. Mycological Progress 15: 811–825. 10.1007/s11557-016-1211-y

[B46] Qin WT, Zhuang WY (2016b) Seven wood-inhabiting new species of the genus *Trichoderma* (Fungi, Ascomycota) in Viride clade. Scientific Reports 6: 27074. 10.1038/srep27074 PMC4888246 27245694

[B47] Qin WT, Zhuang WY (2017) Seven new species of *Trichoderma* (Hypocreales) in the Harzianum and Strictipile clades. Phytotaxa 305(3): 121–139. 10.11646/phytotaxa.305.3.1

[B48] Rashad YM, Shabana YM, Natey. B, Sleem. MM, Hafez. M, Abd-ElGawad AM, Deng Q, Deng JX (2025) *Trichoderma* biodiversity from Egypt and a new *Trichoderma* species, *Trichoderma egyptiacum* sp. nov. (Hypocreaceae, Hypocreales). Mycological Progress 24: 33. 10.1007/s11557-025-02052-9

[B49] Rocha MG, Brilhante RN, Paixäo G, De Oliveira J, Pereira V, De Lima-Neto R, Maia Castelo-Branco DSC, Cordeiro RA, Costa Sidrim J (2019) Chlamydoconidium-producing *Trichophyton tonsurans*: Atypical morphological features of strains causing tinea capitis in Ceará, Brazil. Asian Pacific Journal of Tropical Medicine 12: 380. 10.4103/1995-7645.262567

[B50] Ronquist F, Teslenko M, Van Der Mark P, Ayres DL, Darling A, Höhna S, Larget B, Liu L, Suchard MA, Huelsenbeck JP (2012) MrBayes 3.2: Efficient bayesian phylogenetic inference and model choice across a large model space. Systematic Biology 61: 539–542. 10.1093/sysbio/sys029 PMC3329765 22357727

[B51] Saccardo P, Roumeguère C (1885) Fungi Algerienses, Tahitenses et Gallici. Revue Mycologique 7: 158–161.

[B52] Samuels GJ, Ismaiel A, De Souza J, Chaverri P (2012) *Trichoderma stromaticum* and its overseas relatives. Mycological Progress 11: 215–254. 10.1007/s11557-011-0743-4

[B53] Schalamun M, Schmoll M (2022) *Trichoderma* – genomes and genomics as treasure troves for research towards biology, biotechnology and agriculture. Frontiers in Fungal Biology 3: 1002161. 10.3389/ffunb.2022.1002161 PMC10512326 37746224

[B54] Shao Y, Gu S, Peng H, Zhang L, Li S, Berendsen RL, Yang T, Dong C, Wei Z, Xu Y, Shen Q (2025) Synergic interactions between *Trichoderma* and the soil microbiomes improve plant iron availability and growth. npj Biofilms and Microbiomes 11: 56. 10.1038/s41522-025-00684-z PMC11978894 40199867

[B55] Sousa TF, Vieira Reça BNP, Castro GS, Da Silva IJS, Caniato FF, De Araújo Júnior MB, Yamagishi MEB, Koolen HHF, Bataglion GA, Hanada RE, Da Silva GF (2023) *Trichoderma agriamazonicum* sp. nov. (Hypocreaceae), a new ally in the control of phytopathogens. Microbiological Research 275: 127469. 10.1016/j.micres.2023.127469 37543005

[B56] Sun J, Pei Y, Li E, Li W, Hyde KD, Yin WB, Liu X (2016) A new species of *Trichoderma hypoxylon* harbours abundant secondary metabolites. Scientific Reports 6: 37369. 10.1038/srep37369 PMC5116760 27869187

[B57] Tan QL, Zhang YX, Zhou T, Xiong YR, Huang QR, Liu GH, You CP, Xiong YL, Manawasinghe I (2025) Endophytic *Trichoderma* (Sordariomycetes, Hypocreaceae) species associated with *Citrus* in Guangdong province, China. New Zealand Journal of Botany 63: 251–277. 10.1080/0028825x.2024.2445352

[B58] Thokala P, Kamil D, Toppo R, Choudhary SP (2021) *Trichoderma dumbbelliforme* sp. nov. an undescribed fungus of order Hypocreales from India. Phytotaxa 520: 285–295. 10.11646/phytotaxa.520.3.8

[B59] Wei YH, Tzean SS, Hsieh SY (2024) *Trichoderma changiae* (Hypocreales), a new species isolated from a native orchid in Taiwan. Phytotaxa 658: 261–270. 10.11646/phytotaxa.658.3.4

[B60] Ye CW, Jing TT, Sha YR, Mo MH, Yu ZF (2023) Two new *Trichoderma* species (Hypocreales, Hypocreaceae) isolated from decaying tubers of *Gastrodia elate*. MycoKeys 99: 187–207. 10.3897/mycokeys.99.109404 PMC10504636 37719304

[B61] Zheng H, Qiao M, Lv YF, Du X, Zhang KQ, Yu ZF (2021) New species of *Trichoderma* isolated as endophytes and saprobes from southwest China. Journal of Fungi 7: 467. 10.3390/jof7060467 PMC8230185 34207925

[B62] Zeng XY, Yuan XX, Peng KQ, Pan YT, Tan TJ, Wu N, Tian FH (2022) Taxonomy and control of *Trichoderma hymenopellicola* sp. nov. responsible for the first green mold disease on *Hymenopellis raphanipes*. Frontiers in Microbiology 13: 991987. 10.3389/fmicb.2022.991987 PMC9559395 36246254

[B63] Zeng ZQ, Wang XH, Zhuang WY (2024) New species and new Chinese record of Hypocreaceae from China. Phytotaxa 650: 93–102. 10.11646/phytotaxa.650.1.8

[B64] Zhang GZ, Yang HT, Zhang XJ, Zhou FY, Wu XQ, Xie XY, Zhao XY, Zhou HZ (2022) Five new species of *Trichoderma* from moist soils in China. MycoKeys 87: 133–157. 10.3897/mycokeys.87.76085 PMC8873192 35221753

[B65] Zhao R, Chen KY, Mao LJ, Zhang CL (2025) Eleven new species of *Trichoderma* (Hypocreaceae, Hypocreales) from China. Mycology 16: 180–209. 10.1080/21501203.2024.2330400 PMC11899217 40083403

[B66] Zhao R, Mao LJ, Zhang CL (2023) Three new species of *Trichoderma* (Hypocreales, Hypocreaceae) from soils in China. MycoKeys 97: 21–40. 10.3897/mycokeys.97.101635 PMC10170311 37181496

[B67] Zhao S, Zhang T, Hasunuma T, Kondo A, Zhao XQ, Feng JX (2024) Every road leads to Rome: diverse biosynthetic regulation of plant cell wall-degrading enzymes in filamentous fungi *Penicillium oxalicum* and *Trichoderma reesei*. Critical Reviews in Biotechnology 44: 1241–1261. 10.1080/07388551.2023.2280810 38035670

[B68] Zhu L, Chen Y, Ni W, Zeng J, Li X, Hu C, Li L (2024) The degradation of polyethylene by *Trichoderma* and its impact on soil organic carbon. Agriculture 14: 1821. 10.3390/agriculture14101821

[B69] Zhu ZX, Xu HX, Zhuang WY, Li Y (2017) Two new green-spored species of *Trichoderma* (Sordariomycetes, Ascomycota) and their phylogenetic positions. MycoKeys 26: 61–75. 10.3897/mycokeys.26.14919

[B70] Zhu ZX, Zhuang WY (2015) Three new species of *Trichoderma* with hyaline ascospores from China. Mycologia 107: 328–345. 10.3852/14-141 25572101

[B71] Zimand G, Elad Y, Chet I (1996) Effect of *Trichoderma harzianum* on *Botrytis cinerea* pathogenicity. Phytopathology 86: 1255–1260. 10.1094/phyto-86-1255

