# Supplementary figures and images for: Taxonomy and phylogeny of five novel Trichoderma (Hypocreaceae, Hypocreales) species isolated from decaying wood in China
Source: MycoKeys. 2026 May 27;133:67–102. doi: 10.3897/mycokeys.133.187172 (PMC13234563; doi:10.3897/mycokeys.133.187172)

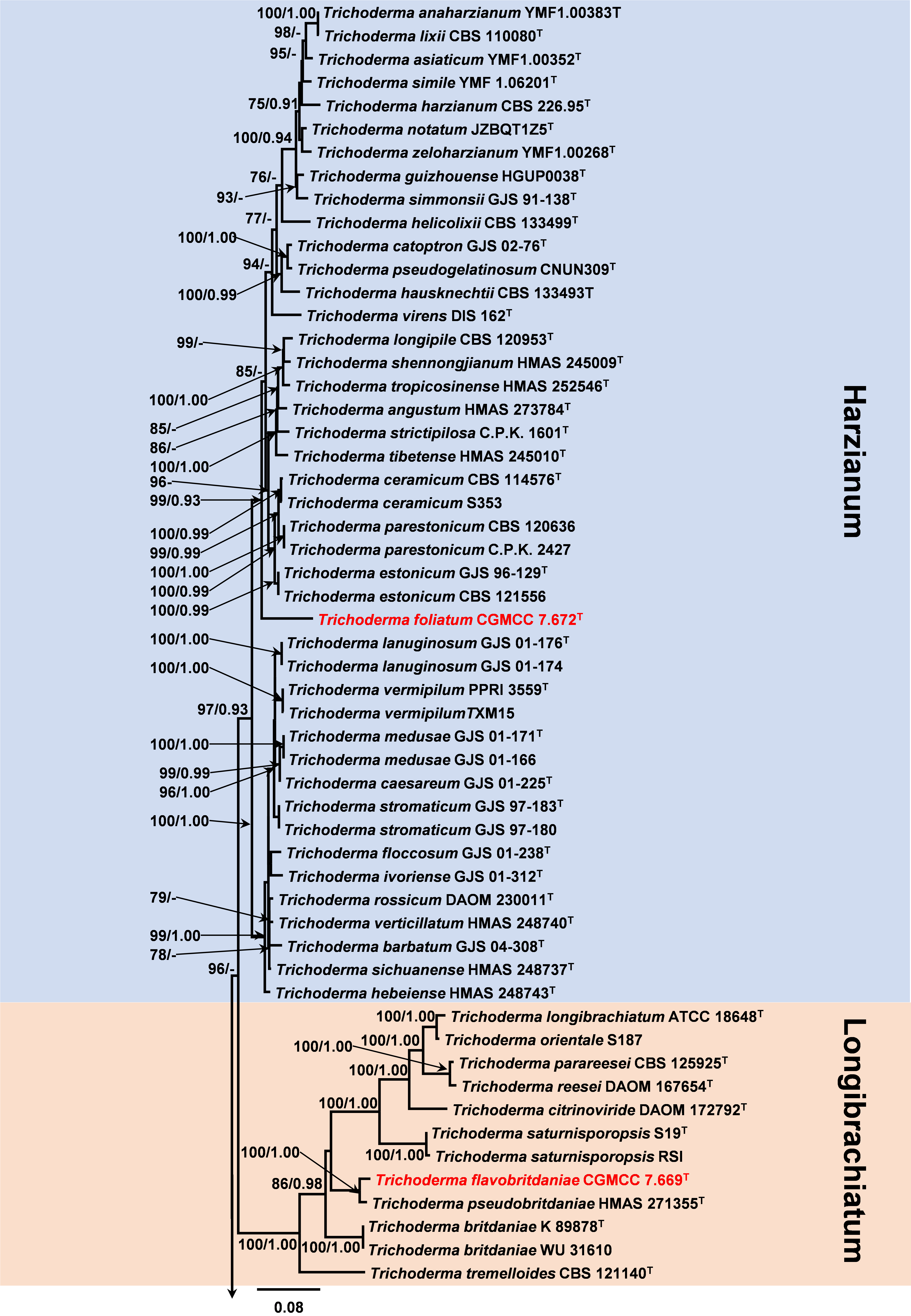

Supplement: Supplementary material 1 — Phylogenetic trees, based on the Maximum Likelihood analysis of the rpb2 dataset [file mycokeys-133-067-s001.zip › 187172_0R-1-A_Supplementary_material_1_Phylogenetic_tree_based_on_the_Maximum_Likelihood_analysis_of_the_rpb2_dataset_MLBP_above_75_right_are_indicat.png]

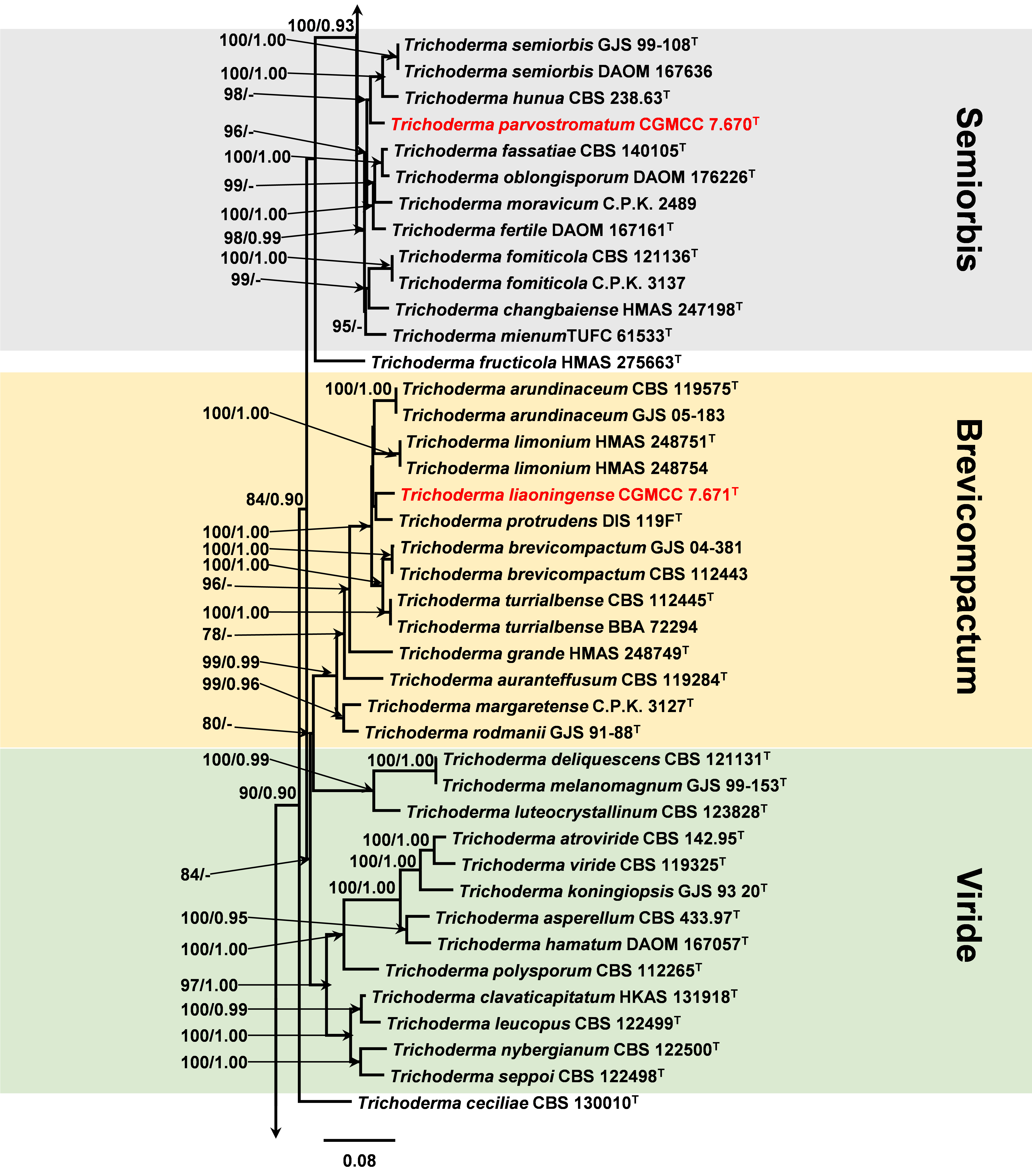

Supplement: Supplementary material 1 — Phylogenetic trees, based on the Maximum Likelihood analysis of the rpb2 dataset [file mycokeys-133-067-s001.zip › 187172_0R-1-A_Supplementary_material_1_Continued_nbsp;.png]

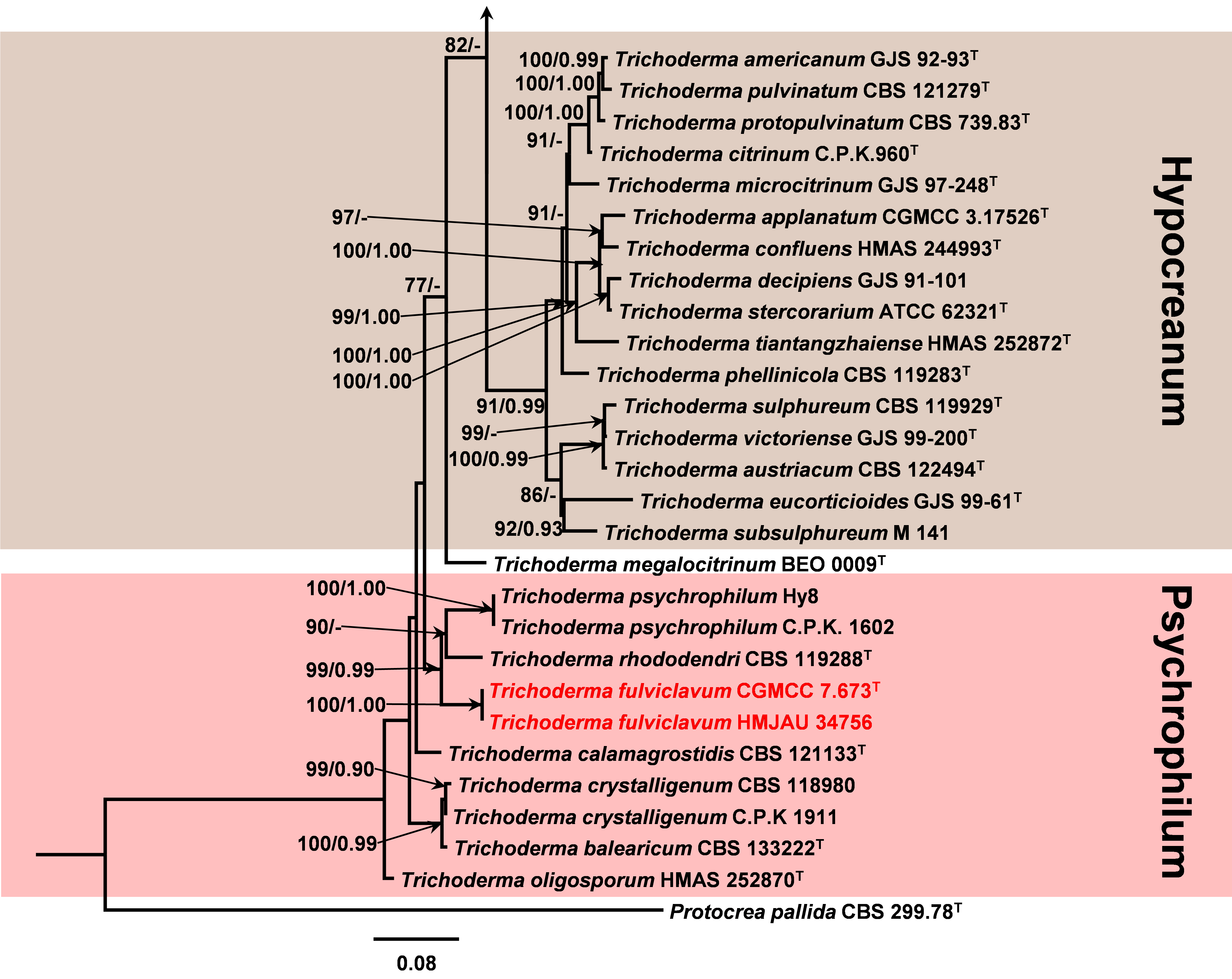

Supplement: Supplementary material 1 — Phylogenetic trees, based on the Maximum Likelihood analysis of the rpb2 dataset [file mycokeys-133-067-s001.zip › 187172_0R-1-A_Supplementary_material_1_Continued_2.png]

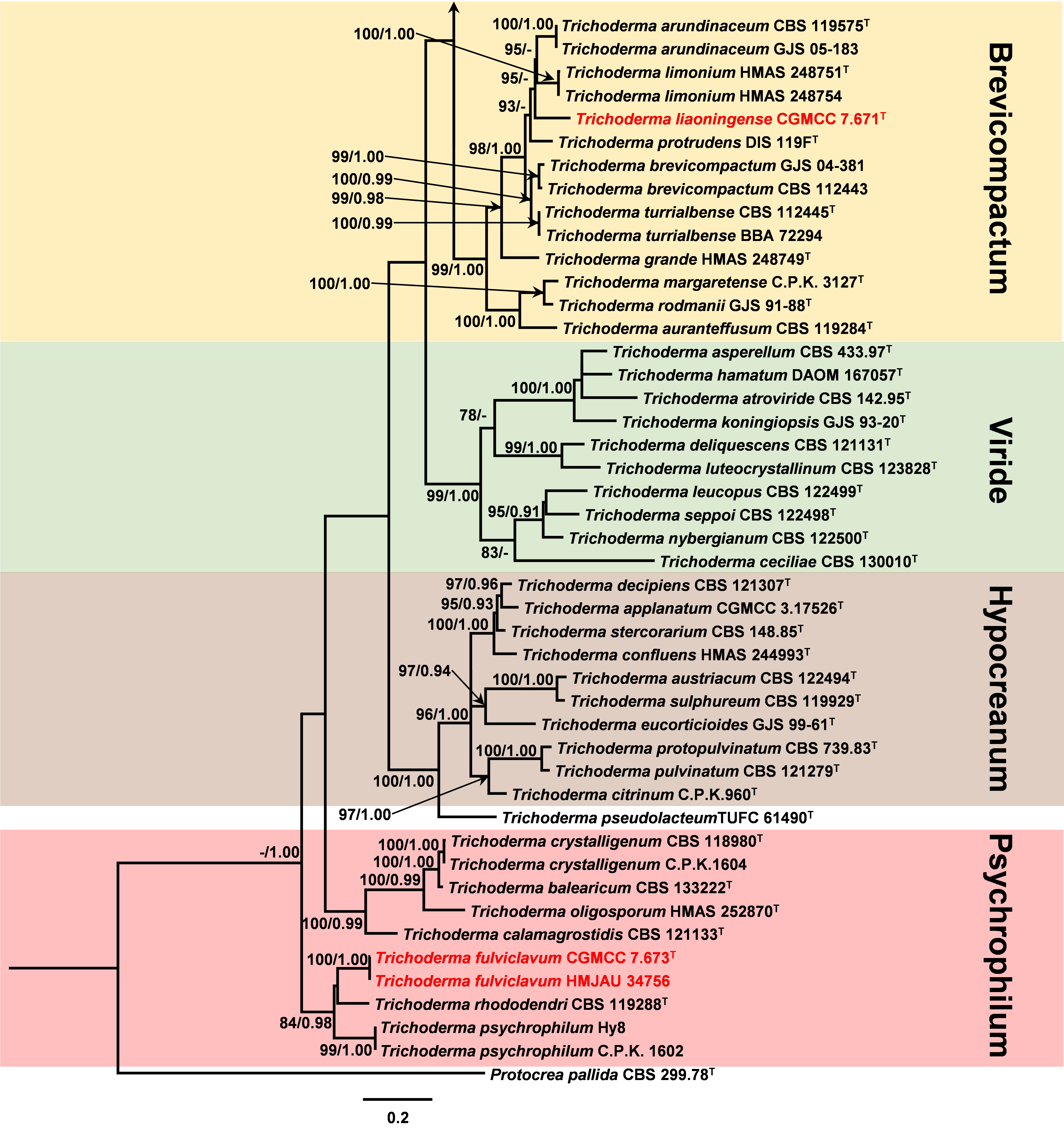

Supplement: Supplementary material 2 — Phylogenetic tree, based on the Maximum Likelihood analysis of the tef1-α dataset [file mycokeys-133-067-s002.zip › 187172_0R-1-A_Supplementary_material_2_Continued_nbsp;.png]

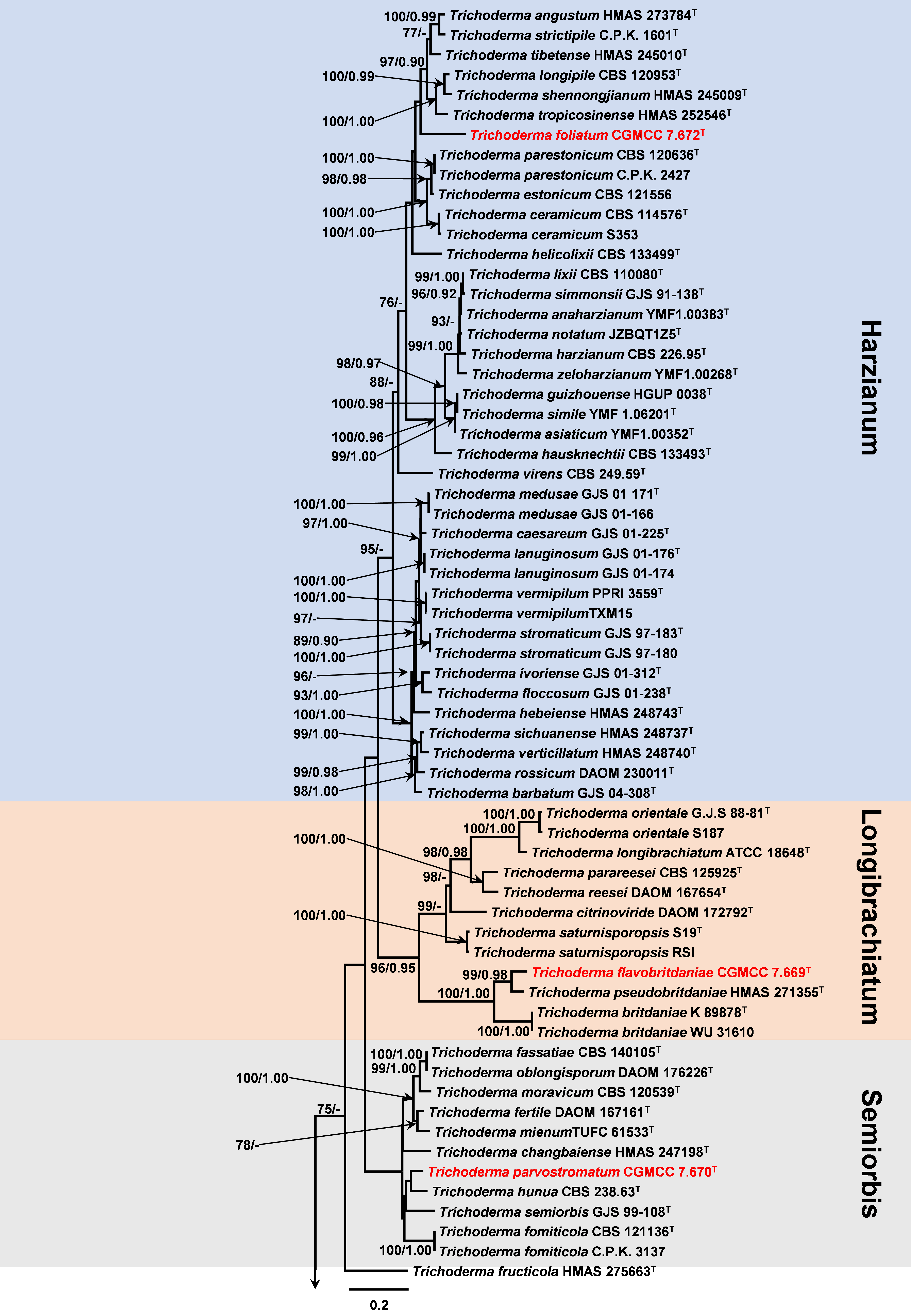

Supplement: Supplementary material 2 — Phylogenetic tree, based on the Maximum Likelihood analysis of the tef1-α dataset [file mycokeys-133-067-s002.zip › 187172_0R-1-A_Supplementary_material_2_Phylogenetic_tree_based_on_the_Maximum_Likelihood_analysis_of_the_tef1-_alpha;_dataset_MLBP_above_75_right_ar.png]
